# Marginal semiparametric accelerated failure time cure model for clustered survival data

**DOI:** 10.1177/09622802241295335

**Published:** 2024-12-10

**Authors:** Yi Niu, Duze Fan, Jie Ding, Yingwei Peng

**Affiliations:** 1 School of Mathematical Sciences, 12399 Dalian University of Technology, Dalian, Liaoning, China; 2 Department of Public Health Sciences, Queen’s University, Kingston, ON, Canada; 3 Department of Mathematics and Statistics, Queen’s University, Kingston, ON, Canada

**Keywords:** Clustered survival data, mixture cure model, efficiency, accelerated failure time model, marginal method, generalized estimating equation

## Abstract

The semiparametric accelerated failure time mixture cure model is an appealing alternative to the proportional hazards mixture cure model in analyzing failure time data with long-term survivors. However, this model was only proposed for independent survival data and it has not been extended to clustered or correlated survival data, partly due to the complexity of the estimation method for the model. In this paper, we consider a marginal semiparametric accelerated failure time mixture cure model for clustered right-censored failure time data with a potential cure fraction. We overcome the complexity of the existing semiparametric method by proposing a generalized estimating equations approach based on the expectation–maximization algorithm to estimate the regression parameters in the model. The correlation structures within clusters are modeled by working correlation matrices in the proposed generalized estimating equations. The large sample properties of the regression estimators are established. Numerical studies demonstrate that the proposed estimation method is easy to use and robust to the misspecification of working matrices and that higher efficiency is achieved when the working correlation structure is closer to the true correlation structure. We apply the proposed model and estimation method to a contralateral breast cancer study and reveal new insights when the potential correlation between patients is taken into account.

## 1. Introduction

Many studies involving time-to-event analysis have a fraction of study subjects who will not experience the event of interest even after an extended follow-up. They are often deemed as cured or long-term survivors who are immune or non-susceptible to the event. For instance, only 5% to 50% of patients with head and neck cancer^1^ experienced local recurrences, and many patients were free of symptoms of cancer at the end of the sufficiently long observation period and can be considered cured. Due to the existence of cured subjects, using classical survival models, such as Cox’s proportional hazards (PH) model, for such data, can result in biased estimates and information loss, and cure models^2,3^ that have been developed to take the cured subjects into account should be considered.

In addition to potentially cured subjects, clustered survival times are often observed in the studies. Clustering may occur when there are multiple events from one subject^4^ or multiple subjects from the same family or hospital,^5^ and the times to the event of interest from the same cluster tend to be correlated due to shared genetic or other common environments.

To appropriately account for the correlation in a cluster, the two most studied approaches are marginal models and frailty models. The marginal models focus on the population average on the marginals of the joint distribution of data from one cluster. The correlation within a cluster is either estimated by a working correlation or treated as a nuisance parameter in the marginal models. Alternatively, frailty models explicitly formulate the underlying dependence structure by random effects or frailties, and the failure times are assumed to be independent conditional on the unobservable frailty. These models have been studied extensively in the literature. For example, Rubio and Drikvandi^6^ developed a novel parametric mixed-effects general hazard model for the analysis of clustered survival data. The heterogeneity between clusters is modeled via the incorporation of random effects into a hazard-based regression model. Chiou et al.^7^ considered a semiparametric accelerated failure time (AFT) model for clustered failure times from stratified random sampling and proposed weighted rank-based estimating equations for fitting the model with the induced smoothing approach. The generalized estimating equations (GEE) method^8^ has been adopted in both the marginal AFT models^9,10^ and the marginal PH models.^11,12^

To model clustered survival data with a cured fraction, the marginal mixture cure model is often assumed and a robust variance estimation is used for inference.^5,13^ The PH assumption was considered for survival times among uncured subjects in the marginal models. This approach was further generalized with a transformation model for survival times among uncured subjects^14,15^ and with GEE to allow for more efficient estimation.^16–18^ Other approaches for modeling clustered survival data were also considered in cure models, including the random effects/frailty approach^19–23^ and the copula approach.^24–27^

The existing models for clustered/correlated survival time with a cured fraction are largely based on the PH assumption when modeling the effects of covariates on the survival time of uncured subjects. Although the PH assumption is widely used in modeling censored survival data, it is not an assumption that is easier to satisfy in practice than other assumptions. It also suffers from difficulty in interpreting the estimated effects. Researchers will benefit from having more than one analytic technique at their disposal when the PH assumption is not appropriate.

One alternative assumption to the PH assumption is the AFT assumption. The attractive feature of the AFT model is that the effects of covariates are modeled directly on the expected value of the survival time, making the interpretation more intuitive and straightforward than the effects from the PH assumption.^28^ The AFT model also enjoys other desirable properties, including collapsibility, that are not exhibited by a PH model. The collapsibility makes the AFT model particularly attractive when quantifying confounding effects^29^ and mediation effects of covariates^30,31^ in causal inference.

The AFT assumption has been considered in models for survival data with a cure fraction, including parametric AFT mixture cure models^32,33^ and semiparametric AFT mixture cure models.^34–41^ However, we are not aware of any existing work on modeling clustered survival data with a cured fraction using the AFT assumption, particularly under semiparametric models. This may be due to the challenges in extending the existing semiparametric estimation methods to clustered survival data. It motivates us to consider AFT assumption-based cure models for clustered survival time with a cured fraction. This work is important because it fills the gap in the literature and provides useful alternatives to the PH-based models for clustered survival data with a cured fraction. The interpretation of covariate effects in the AFT assumption-based models is more straightforward and intuitive, and the models are more suitable for future work in causal inference.

In this paper, we propose a marginal semiparametric AFT mixture cure model for clustered survival time data with a cured fraction. An estimating equation approach is employed to estimate the regression parameters in the model with flexible working correlation structures for cure statuses and for the survival times of uncured subjects within clusters. The paper is organized as follows. Section 2 introduces the marginal semiparametric AFT mixture cure model for clustered survival data with a cure fraction. A semiparametric estimation method of the model based on a set of GEE is presented and the asymptotic properties of the estimators are investigated in this section. A simulation study is conducted in Section 3 to evaluate the finite sample performance of the proposed estimation method. The proposed model and the estimation method are applied to contralateral breast cancer data in Section 4. Conclusions and discussions are presented in Section 5.

## 2. Marginal AFT mixture cure model

Let ${\tilde{T}}_{ij}$ and $C_{ij}$ be the failure time and censoring time of the j th subject in the i th cluster, where i=1,2,…,K , K is the number of clusters, $j=1,2,\ldots ,n_{i}$ , and $n_{i}$ is the number of subjects in the i th cluster. The total number of subjects in all clusters is $N=\sum_{i=1}^{K}n_{i}$ . Denote $T_{ij}=\min({\tilde{T}}_{ij},C_{ij})$ as the observed failure time and $\delta_{ij}=I\{{\tilde{T}}_{ij}\leq C_{ij}\}$ as the censoring indicator. Let $Z_{ij}$ and $X_{ij}$ denote a $p_{Z}\times 1$ vector of covariates and a $p_{X}\times 1$ vector of covariates, respectively. Given $Z_{ij}$ and $X_{ij}$ , the censoring time $C_{ij}$ is assumed to be independent of ${\tilde{T}}_{ij}$ . Let $\omega_{ij}$ be the cure status of the j th subject in the i th cluster, where $\omega_{ij}=0$ if the subject is cured and $\omega_{ij}=1$ if not. We further assume that given $X_{ij}$ , $Z_{ij}$ , $X_{i^{\prime }j^{\prime }}$ , and $Z_{i^{\prime }j^{\prime }}$ , ${\tilde{T}}_{ij}|(\omega_{ij}=1)$ and ${\tilde{T}}_{ij^{\prime }}|(\omega_{ij^{\prime }}=1)$ , and $\omega_{ij}$ and $\omega_{ij^{\prime }}$ for $j\neq j^{\prime }$ are correlated, respectively. However, for $i\neq i^{\prime }$ , ${\tilde{T}}_{ij}|(\omega_{ij}=1)$ and ${\tilde{T}}_{i^{\prime }j^{\prime }}|(\omega_{i^{\prime }j^{\prime }}=1)$ , and $\omega_{ij}$ and $\omega_{i^{\prime }j^{\prime }}$ are assumed independent, separately. For cured subjects with $\omega_{ij}=0$ , we assume ${\tilde{T}}_{ij}=\infty$ (a finite value is possible as long as it is beyond the support of the distribution of ${\tilde{T}}_{ij}|(w_{ij}=1)$ ). It is clear $\omega_{ij}=1$ if $\delta_{ij}=1$ , and $\omega_{ij}$ is usually unknown if $\delta_{ij}=0$ .

Let $S(t|X_{ij},Z_{ij})$ denote the survival function of ${\tilde{T}}_{ij}$ . We propose the following semiparametric marginal AFT mixture cure model for the data above:

(1) $$S(t|X_{ij},Z_{ij})=P({\tilde{T}}_{ij}>t|X_{ij},Z_{ij})=1-\pi (Z_{ij})+\pi (Z_{ij})S_{u}(t|X_{ij})$$

where $\pi (Z_{ij})$ (referred to as the incidence part of the model) is the uncured probability specified as

(2) $$\pi (Z_{ij})=P(\omega_{ij}=1|Z_{ij})=\frac{\exp\;(\gamma^{\prime }Z_{ij})}{1+\exp\;(\gamma^{\prime }Z_{ij})}$$

γ is a $(p_{Z}+1)\times 1$ vector of unknown parameters (the intercept is included), $S_{u}(t|X_{ij})$ is the survival function of ${\tilde{T}}_{ij}>t|(\omega_{ij}=1)$ (referred to as the latency part of the model), which is assumed to follow the AFT model:

(3) $$\log\;{\tilde{T}}_{ij}=\beta^{\prime }X_{ij}+\varepsilon_{ij}$$

where β is a $p_{X}\times 1$ vector of unknown parameters (the intercept is excluded), and $\varepsilon_{ij}$ is an error term with an unspecified survival function $S_{\varepsilon }(\cdot )$ . It is clear that $S_{u}(t|X_{ij})=S_{\varepsilon }(\log\;t-\beta^{\prime }X_{ij})$ .

If we assume all $\omega_{ij}$ are known and ignore the correlation within clusters, the likelihood function for the marginal survival model (1) based on available data $\left\{(t_{ij},\delta_{ij},X_{ij},Z_{ij},\omega_{ij}),i=1,2,\ldots ,K;j=1,2,\ldots ,n_{i}\right\}$ is

$$\begin{array}{rl} \ell_{c}\left(\beta ,\gamma ,S_{\varepsilon }\left(\cdot \right)\right) & =\sum_{i=1}^{K}\sum_{j=1}^{n_{i}}\{\omega_{ij}\log\;\pi \left(Z_{ij}\right)+(1-\omega_{ij})\log\;\left[1-\pi \left(Z_{ij}\right)\right]\}\\ & \;+\sum_{i=1}^{K}\sum_{j=1}^{n_{i}}\omega_{ij}\left\{\delta_{ij}\log\;f_{\varepsilon }\left(\varepsilon_{ij}\left(\beta \right)\right)+(1-\delta_{ij})\log\;S_{\varepsilon }\left(\varepsilon_{ij}\left(\beta \right)\right)\right\} \end{array}$$

where $\varepsilon_{ij}(\beta )=\log\;t_{ij}-\beta^{\prime }X_{ij}$ and $f_{\varepsilon }(\cdot )$ is the corresponding density function of $S_{\varepsilon }(\cdot )$ . The first term involves γ whereas the second term involves β and $S_{\varepsilon }$ . The expectation–maximization (EM) algorithm can be employed to maximize the likelihood function. Let ${\hat{\gamma }}^{\left(m\right)}$ , ${\hat{\beta }}^{\left(m\right)}$ , and ${\hat{S}}_{\varepsilon }^{\left(m\right)}(\cdot )$ denote the estimates of γ , β , and $S_{\varepsilon }(\cdot )$ , respectively, in the m th iteration of the algorithm. The E-step in the next iteration of the EM algorithm calculates the conditional expectation of $\ell_{c}$ with respect to $\omega_{ij}$ , which is equivalent to replacing $\omega_{ij}$ in $\ell_{c}$ with its posterior expectation conditional on observed data and the current estimates:

(4) $$\begin{array}{rl} g_{ij}^{\left(m\right)} & =E(\omega_{ij}|t_{ij},\delta_{ij},X_{ij},Z_{ij},{\hat{\gamma }}^{\left(m\right)},{\hat{\beta }}^{\left(m\right)},{\hat{S}}_{\varepsilon }^{\left(m\right)}(\cdot ))\\ & =\delta_{ij}+{\frac{\left(1-\delta_{ij}\right)\pi \left(Z_{ij}\right)S_{\varepsilon }\left(\varepsilon_{ij}\left(\beta \right)\right)}{1-\pi \left(Z_{ij}\right)+\pi \left(Z_{ij}\right)S_{\varepsilon }\left(\varepsilon_{ij}\left(\beta \right)\right)}|}_{\left(\gamma ,\beta ,S_{\varepsilon }(\cdot ))=({\hat{\gamma }}^{\left(m\right)},{\hat{\beta }}^{\left(m\right)},{\hat{S}}_{\varepsilon }^{\left(m\right)}(\cdot )\right)} \end{array}$$

The M-step solves the following estimating equations with respect to γ and β separately to obtain ${\hat{\gamma }}^{\left(m+1\right)}$ and ${\hat{\beta }}^{\left(m+1\right)}$

(5) $$\begin{array}{rl} \sum_{i=1}^{K}\sum_{j=1}^{n_{i}}{\left(\frac{\partial \pi \left(Z_{ij}\right)}{\partial \gamma }\right)}^{\prime }{\left[\pi \left(Z_{ij}\right)\left(1-\pi \left(Z_{ij}\right)\right)\right]}^{-1}\left(g_{ij}^{\left(m\right)}-\pi \left(Z_{ij}\right)\right) & =0 \end{array}$$

(6) $$\begin{array}{rl} \sum_{i=1}^{K}\sum_{j=1}^{n_{i}}g_{ij}^{\left(m\right)}X_{ij}\left\{-\delta_{ij}\frac{(df_{\varepsilon }/d\varepsilon )\left(\varepsilon_{ij}(\beta )\right)}{f_{\varepsilon }\left(\varepsilon_{ij}(\beta )\right)}+\left(1-\delta_{ij}\right)\frac{f_{\varepsilon }\left(\varepsilon_{ij}(\beta )\right)}{S_{\varepsilon }\left(\varepsilon_{ij}(\beta )\right)}\right\} & =0 \end{array}$$

Due to the unspecified baseline distribution, the estimating equation (6) cannot be solved directly. Following Ritov,^42^ we replace $-(df_{\varepsilon }/d\varepsilon )(\varepsilon )/f_{\varepsilon }(\varepsilon )$ with a score function η(ε)=ε and center $X_{ij}$ to account for the unknown intercept term in (6) to obtain the following estimating equation:

(7) $$\sum_{i=1}^{K}\sum_{j=1}^{n_{i}}g_{ij}^{\left(m\right)}(X_{ij}-\bar{X})\left\{\delta_{ij}\varepsilon_{ij}(\beta )+\left(1-\delta_{ij}\right)\frac{\int_{\varepsilon_{ij}(\beta )}^{+\infty }udF_{\varepsilon }(u)}{S_{\varepsilon }\left(\varepsilon_{ij}(\beta )\right)}\right\}=0$$

where $\bar{X}=N^{-1}\sum_{i=1}^{K}\sum_{j=1}^{n_{i}}X_{ij}$ and $F_{\varepsilon }(\cdot )$ is the corresponding distribution function of $S_{\varepsilon }(\cdot )$ . To obtain ${\hat{\beta }}^{\left(m+1\right)}$ from this estimating equation, an estimate of $S_{\varepsilon }(y)$ is needed. It can be nonparametrically estimated by Zhang and Peng^35^

(8) $${\hat{S}}_{\varepsilon }^{\left(m+1\right)}(y)=\exp\;\left(-\sum_{s:\tau_{s}<y}\frac{d_{s}}{\sum_{(i,j)\in R\left(\tau_{s}\right)}g_{ij}^{\left(m\right)}}\right)$$

where $\tau_{1},\ldots ,\tau_{k}$ are the distinct uncensored failure residuals $\varepsilon_{ij}(\beta^{\left(m\right)})$ , $d_{s}=\sum_{i=1}^{K}\sum_{j=1}^{n_{i}}\delta_{ij}I\{\varepsilon_{ij}(\beta^{\left(m\right)})=\tau_{s}\}$ denotes the number of subjects with failure at $\tau_{s}$ , and $R(\tau_{s})=\{(i,j):\varepsilon_{ij}(\beta^{\left(m\right)})\geq \tau_{s},i=1,2,\ldots ,K,j=1,2,\ldots ,n_{i}\}$ is the risk set at $\tau_{s}$ . To enhance the identifiability of the parameter estimation, we also set ${\hat{S}}_{\varepsilon }^{\left(m\right)}(y)=0$ when $y>\tau_{k}$ as in other semiparametric cure model estimation methods.^2^

To take the potential correlation within clusters into account in the estimation, we consider incorporating working correlation matrices into the estimating equations (5) and (7). Let $Q_{i}^{\left(1\right)}$ and $Q_{i}^{\left(2\right)}$ be the $n_{i}\times n_{i}$ working correlation matrices that approximate the correlation structure among $\omega_{ij}$ s and the correlation structure among $\log\;{\tilde{T}}_{ij}|(\omega_{ij}=1)$ s, respectively, within the i th cluster. Following Liang and Zeger,^8^ we propose the following GEE for γ that includes the working correlation matrix $Q_{i}^{\left(1\right)}$ for $\omega_{ij}$ :

(9) $$U(\gamma )=\sum_{i=1}^{K}{\left\{\frac{\partial \pi_{i}}{\partial \gamma }\right\}}^{\prime }{\left\{A_{i}^{1/2}Q_{i}^{\left(1\right)}A_{i}^{1/2}\phi_{1}\right\}}^{-1}\left\{g_{i}-\pi_{i}\right\}=0$$

where $\pi_{i}=\{\pi (Z_{i1}),\ldots ,\pi (Z_{in_{i}}){\}}^{\prime }$ , $A_{i}$ is a diagonal matrix with diagonal elements $\pi (Z_{i1})[1-\pi (Z_{i1})],\ldots ,\pi (Z_{in_{i}})[1-\pi (Z_{in_{i}})]$ , $g_{i}=(g_{i1}^{\left(m\right)},g_{i2}^{\left(m\right)},\ldots ,g_{in_{i}}^{\left(m\right)}{)}^{\prime }$ , and $\phi_{1}$ is the scale parameter to accommodate potential over- or under-dispersion. When there is no correlation within clusters and $\phi_{1}=1$ , $Q_{i}^{\left(1\right)}$ reduces to an identity matrix and equation (9) reduces to equation (5).

To develop the GEE for β , we rewrite equation (7) as follows:

$$\begin{array}{rl} & \sum_{i=1}^{K}\sum_{j=1}^{n_{i}}g_{ij}^{\left(m\right)}(X_{ij}-\bar{X})\left\{\delta_{ij}\log\;t_{ij}+\left(1-\delta_{ij}\right)\left[\frac{\int_{\varepsilon_{ij}(\beta )}^{+\infty }udF_{\varepsilon }(u)}{S_{\varepsilon }\left(\varepsilon_{ij}(\beta )\right)}+\beta^{\prime }X_{ij}\right]-\beta^{\prime }X_{ij}\right\}\\ & \;=\sum_{i=1}^{K}\sum_{j=1}^{n_{i}}g_{ij}^{\left(m\right)}(X_{ij}-\bar{X})\left\{{\hat{y}}_{ij}(\beta )-\beta^{\prime }X_{ij}\right\} \end{array}$$

where

$${\hat{y}}_{ij}(\beta )=\delta_{ij}\log\;t_{ij}+(1-\delta_{ij})\left[\frac{\int_{\varepsilon_{ij}(\beta )}^{+\infty }udF_{\varepsilon }(u)}{S_{\varepsilon }(\varepsilon_{ij}(\beta ))}+\beta^{\prime }X_{ij}\right]$$

which is $\log\;t_{ij}$ if $\delta_{ij}=1$ and is $E(\log\;{\tilde{T}}_{ij}|{\tilde{T}}_{ij}>C_{ij})$ if $\delta_{ij}=0$ . A similar estimating equation was also proposed by Buckley and James.^43^ We propose the following GEE for β that includes the working correlation matrix $Q_{i}^{\left(2\right)}$ :

(10) $$U(\beta )=\sum_{i=1}^{K}{\left(X_{i}-1_{i}{\bar{X}}^{\prime }\right)}^{\prime }{\left\{B_{i}^{1/2}Q_{i}^{\left(2\right)}B_{i}^{1/2}\phi_{2}\right\}}^{-1}G_{i}\left({\hat{Y}}_{i}(\beta )-X_{i}\beta \right)=0$$

where $X_{i}=(X_{i1},X_{i2},\ldots ,X_{in_{i}}{)}^{\prime }$ , $1_{i}$ is an $n_{i}\times 1$ vector of 1s, $B_{i}$ is an $n_{i}\times n_{i}$ diagonal variance matrix with element $\sigma_{\hat{Y}(\beta )}^{2}$ , $G_{i}$ is the diagonal matrix with $g_{i}$ , ${\hat{Y}}_{i}(\beta )=({\hat{y}}_{i1}(\beta ),\ldots ,{\hat{y}}_{in_{i}}(\beta ){)}^{\prime }$ , and $\phi_{2}$ is the scale parameter to accommodate potential over- or under-dispersion. When there is no correlation within clusters and $\phi_{2}=1$ , $Q_{i}^{\left(2\right)}$ reduces to the identity matrix and the GEE (10) reduces to equation (7). Similar to (7), equation (10) relies on the unknown $S_{\varepsilon }(\cdot )$ . We suggest to use (8) to estimate $S_{\varepsilon }(\cdot )$ . It is a consistent estimate but may not be efficient. In the simulation, we demonstrate that it still allows efficient estimation of β from (10).

Using the generalized estimation equations (9) and (10) also requires a full specification of $Q_{i}^{\left(1\right)}$ and $Q_{i}^{\left(2\right)}$ . Depending on the nature of the correlation structure, the working correlation matrices $Q_{i}^{\left(1\right)}$ and $Q_{i}^{\left(2\right)}$ can be specified in some special structures. The common working correlation structures are the independent correlation structure, the exchangeable correlation structure (equicorrelated, compound symmetric), and the first-order autoregressive correlation structure. Although the exchangeable correlation structure may be popular for clustered data, the first-order autoregressive correlation structure or other more complicated correlation structures could be considered for clustered data if there is a temporal or spatial distance among the subjects in the cluster that may affect the strength of the correlation. The working correlation matrices are the matrices with the diagonal elements equal to 1, and the off-diagonal elements equal to 0 for the working independent correlation structure, ρ for the exchangeable correlation structure, and $\rho^{|j-j^{\prime }|}$ at the j th row and $j^{\prime }$ th column for the first-order autoregressive correlation structure. Let $\rho_{1}$ and $\rho_{2}$ be ρ in $Q_{i}^{\left(1\right)}$ and $Q_{i}^{\left(2\right)}$ when $Q_{i}^{\left(1\right)}$ and $Q_{i}^{\left(2\right)}$ are assumed to have the exchangeable correlation or the first-order autoregressive correlation structures. Consistent estimates of $\rho_{1}$ and $\phi_{1}$ can be obtained based on the standardized Pearson residuals while consistent estimates of $\rho_{2}$ and $\phi_{2}$ can be obtained based on the residual of the log-linear model. Specifically, if $Q_{i}^{\left(1\right)}$ and $Q_{i}^{\left(2\right)}$ are in the exchangeable correlation structure, then

(11) $$\begin{array}{rl} {\hat{\rho }}_{1} & ={\hat{\phi }}_{1}^{-1}\sum_{i=1}^{K}\sum_{j>j^{\prime }}{\hat{r}}_{ij}^{\left(1\right)}{\hat{r}}_{ij^{\prime }}^{\left(1\right)}/\left\{\sum_{i=1}^{K}\frac{1}{2}n_{i}\left(n_{i}-1\right)-p_{Z}-1\right\} \end{array}$$

(12) $$\begin{array}{rl} {\hat{\rho }}_{2} & ={\hat{\phi }}_{2}^{-1}\sum_{i=1}^{K}\sum_{j>j^{\prime }}{\hat{r}}_{ij}^{\left(2\right)}{\hat{r}}_{ij^{\prime }}^{\left(2\right)}/\left\{\sum_{i=1}^{K}\frac{1}{2}n_{i}\left(n_{i}-1\right)-p_{X}\right\} \end{array}$$

and if $Q_{i}^{\left(1\right)}$ and $Q_{i}^{\left(2\right)}$ are in the first-order autoregressive correlation structure, then

(13) $$\begin{array}{rl} {\hat{\rho }}_{1} & ={\hat{\phi }}_{1}^{-1}\sum_{i=1}^{K}\sum_{j\leq n_{i}-1}{\hat{r}}_{ij}^{\left(1\right)}{\hat{r}}_{i,j+1}^{\left(1\right)}/\left\{\sum_{i=1}^{K}\left(n_{i}-1\right)-p_{Z}-1\right\} \end{array}$$

(14) $$\begin{array}{rl} {\hat{\rho }}_{2} & ={\hat{\phi }}_{2}^{-1}\sum_{i=1}^{K}\sum_{j\leq n_{i}-1}{\hat{r}}_{ij}^{\left(2\right)}{\hat{r}}_{i,j+1}^{\left(2\right)}/\left\{\sum_{i=1}^{K}\left(n_{i}-1\right)-p_{X}\right\} \end{array}$$

where

(15) $$\begin{array}{rl} {\hat{\phi }}_{1} & =\sum_{i=1}^{K}\sum_{j=1}^{n_{i}}{\left\{{\hat{r}}_{ij}^{\left(1\right)}\right\}}^{2}/(N-p_{Z}-1),\\ {\hat{r}}_{ij}^{\left(1\right)} & =\left\{g_{ij}^{\left(m\right)}-\pi (Z_{ij})\right\}/{\left[\pi (Z_{ij})(1-\pi (Z_{ij}))\right]}^{1/2} \end{array}\;\begin{array}{rl} {\hat{\phi }}_{2} & =\sum_{i=1}^{K}\sum_{j=1}^{n_{i}}{\left\{{\hat{r}}_{ij}^{\left(2\right)}\right\}}^{2}/(N-p_{X})\\ {\hat{r}}_{ij}^{\left(2\right)} & ={\hat{y}}_{ij}({\hat{\beta }}^{\left(m\right)})-{\hat{\beta }}^{\left(m\right)}X_{ij} \end{array}$$

The proposed algorithm for the semiparametric marginal AFT mixture cure model can be summarized as follows:

(a) Set initial values $\gamma^{\left(1\right)}$ , $\beta^{\left(1\right)}$ , and $S_{\varepsilon }^{\left(1\right)}(\cdot )$ .
(b) E-Step: Calculate $g_{ij}^{\left(m\right)}$ via (4).
(c) M-Step:
  (i) Calculate the estimate of $S_{\varepsilon }(\cdot )$ using (8).
  (ii) Given the current estimates of γ and β , calculate the residuals and the estimates of $\rho_{1}$ , $\rho_{2}$ , $\phi_{1}$ , and $\phi_{2}$ via (11), (12), (15), or (13) to (15), depending on the working correlation structure used.
  (iii) Update the estimate of γ from (9) and the estimate of β from (10).
  (iv) Repeat steps (ii) and (iii) until convergence. Denote the estimates as $\gamma^{\left(m+1\right)}$ , $\beta^{\left(m+1\right)}$ , and $S_{\varepsilon }^{\left(m+1\right)}(\cdot )$ .
(d) Repeat steps (b) and (c) until the algorithm converges. The stopping criterion is that the sum of the squares of the differences in estimates between two adjacent iterations is below $10^{-4}$ .

Let $\hat{\theta }=({\hat{\gamma }}^{\prime },{\hat{\beta }}^{\prime }{)}^{\prime }$ be the final estimator of $\theta =(\gamma^{\prime },\beta^{\prime }{)}^{\prime }$ . Under certain regularity conditions, we establish the asymptotic properties for $\hat{\theta }$ in the following theorem.

Theorem 1 Let $\theta_{0}=(\gamma_{0}^{\prime },\beta_{0}^{\prime }{)}^{\prime }$ be the true value of θ . Given the consistency of ${\hat{S}}_{\varepsilon }(\cdot )$ , under some regularity conditions provided in the Appendix, we have that

(a) the estimator $\hat{\theta }$ is a consistent estimator of $\theta_{0}$ ;
(b) as K→∞ , $K^{1/2}(\hat{\theta }-\theta_{0})\to N(\text{0},\Sigma )$ in distribution, where $\Sigma =A^{-1}(\theta_{0})V(\theta_{0})\{A^{-1}(\theta_{0}){\}}^{\prime }$ , $A(\theta_{0})=E\{B(\theta_{0})\}-E\{S(\theta_{0})S^{\prime }(\theta_{0})\}$ , $V(\theta_{0})=\Sigma_{i=1}^{K}E\{S_{i}(\theta_{0})\}E\{S_{i}^{\prime }(\theta_{0})\}$ , B(θ)=−∂S(θ)/∂θ , $S(\theta )=(U^{\prime }(\gamma ),U^{\prime }(\beta ){)}^{\prime }$ , $S_{i}(\theta )=(U_{i}^{\prime }(\gamma ),U_{i}^{\prime }(\beta ){)}^{\prime }$ , $U_{i}(\gamma )={\left\{\partial \pi_{i}/\partial \gamma \right\}}^{\prime }\{A_{i}^{1/2}Q_{i}^{\left(1\right)}A_{i}^{1/2}\phi_{1}{\}}^{-1}\{g_{i}-\pi_{i}\}$ , and $U_{i}(\beta )=(X_{i}-1_{i}{\bar{X}}^{\prime }{)}^{\prime }\{B_{i}^{1/2}Q_{i}^{\left(2\right)}B_{i}^{1/2}\phi_{2}{\}}^{-1}G_{i}({\hat{Y}}_{i}(\beta )-X_{i}\beta )$ .

A proof of the theorem is provided in the Appendix. Note that the theorem only provides the asymptotic properties of $\hat{\gamma }$ and $\hat{\beta }$ when other parameter estimates are viewed as fixed. The asymptotic properties of other parameter estimates in the model remain to be worked out. It implies that the variance estimates of $\hat{\gamma }$ and $\hat{\beta }$ based on Σ in the theorem may not be accurate due to the unaccounted variation in other parameter estimates. To obtain better variance estimates for $\hat{\gamma }$ and $\hat{\beta }$ and to obtain variance estimates of other parameter estimates in the model, we consider the bootstrap method.^44^ This method has been adopted by other researchers to deal with the variance estimation in models based on AFT assumption.^9,35,45^ In this method, a bootstrap sample is obtained by sampling clusters with replacement. That is, all observations from one cluster are either included or excluded in one bootstrap sample. Our numerical study in the next section shows that the bootstrap method works well in approximating the standard errors of the parameter estimates in the model.

## 3. Simulation study

We conduct an extensive simulation study to investigate the finite sample performance of the proposed method. Simulated clustered survival data are generated from a mixture cure model with the marginal specified by (1) to (3). Two covariates are considered in $X_{ij}$ and $Z_{ij}$ , one is from the Bernoulli distribution B(1,0.5) , and the other is from the uniform distribution U(−1,1) . For each simulated dataset, the number of clusters and the cluster size $\left(K,n_{i}\right)$ are set as (100,3) and (60,5), separately.

To generate correlated $\omega_{ij}$ s with the marginal model (2), we follow the method by Emrich and Piedmonte.^46^ That is, given the marginal probabilities $\pi (Z_{ij})$ and $\pi (Z_{ij^{\prime }})$ within a cluster, we solve for ${\tilde{\rho }}_{ijj^{\prime }}$ in the following equation:

$$\frac{\Phi \left\{\left[z_{\pi \left(Z_{ij}\right)},z_{\pi (Z_{ij^{\prime }})}\right],{\tilde{\rho }}_{ijj^{\prime }}\right\}-\pi \left(Z_{ij}\right)\pi \left(Z_{ij^{\prime }}\right)}{\{\pi \left(Z_{ij}\right)\pi \left(Z_{ij^{\prime }}\right)\left[1-\pi \left(Z_{ij}\right)\right]\left[1-\pi \left(Z_{ij^{\prime }}\right)\right]{\}}^{1/2}}=\zeta_{ijj^{\prime }}$$

where $\Phi \{\cdot ,{\tilde{\rho }}_{ijj^{\prime }}\}$ is the standard bivariate normal distribution with correlation coefficient ${\tilde{\rho }}_{ijj^{\prime }}$ , $z_{\pi (Z_{ij})}$ is the $\pi (Z_{ij})$ th quantile of the standard normal distribution, $\zeta_{ijj^{\prime }}=1$ for $j=j^{\prime }$ , and $\zeta_{ijj^{\prime }}=\zeta$ for all $j\neq j^{\prime }$ if the exchangeable correlation structure is assumed for $\omega_{ij}$ s and $\zeta_{ijj^{\prime }}=\zeta^{\left|j-j^{\prime }\right|}$ for all $j\neq j^{\prime }$ if the first-order autoregressive correlation structure is assumed for $\omega_{ij}$ s. We then generate $\left(z_{i1},z_{i2},\ldots ,z_{in_{i}}\right)$ from the multivariate normal distribution $N(0,\Sigma_{1i}),$ where $\Sigma_{1i}$ has ${\tilde{\rho }}_{ijj^{\prime }}$ as the matrix elements, and then obtain correlated $\omega_{ij}$ s by $\omega_{ij}=I\{z_{ij}<z_{\pi (Z_{ij})}\}$ . The value of ζ measures the strength of the correlation among the generated $\omega_{ij}$ s within a cluster. The R package mvtBinaryEP is available to produce binary data using the procedure above.

To generate correlated ${\tilde{T}}_{ij}$ s given $\omega_{ij}=1$ in the AFT model (3), we produce $\varepsilon_{i1},\ldots ,\varepsilon_{in_{i}}$ from a multivariate normal distribution $N(0,\Sigma_{2i})$ , where the covariance matrix $\Sigma_{2i}$ has elements defined in a similar way as in $\Sigma_{1i}$ and τ is used to measure the strength of the correlation among $\varepsilon_{ij}$ s within a cluster. Then ${\tilde{T}}_{ij}$ can be obtained following (3). The R package mvtnorm can be used to generate the data.

We set (ζ,τ)=(0.4,0.8) , (0.2,0,5) , and (0,0) to have strong, weak, and no correlation within clusters. We set $\beta =(\beta_{1},\beta_{2}{)}^{\prime }=(0.6,0.9)$ and $\gamma =(\gamma_{0},\gamma_{1},\gamma_{2}{)}^{\prime }=(-0.3,0.6,0.9{)}^{\prime }$ and $(0.3,0.6,0.9{)}^{\prime }$ so that the average cure rate is about 0.5 and 0.35, respectively. The censoring times are noninformative and generated from the uniform distribution U(0,50) which results in a censoring rate of 0.55 when the cure rate is 0.5 and a censoring rate of 0.40 when the cure rate is 0.35. Without loss of the generality, we assume equal cluster sizes and let $n_{i}=n$ and consider (K,n)=(100,3) and (60,5) .

**Table 1.** Bias, Var, Var ${}^{*}$ , CP of 95% confidence intervals of ${\hat{\gamma }}_{0},{\hat{\gamma }}_{1},{\hat{\gamma }}_{2},{\hat{\beta }}_{1},{\hat{\beta }}_{2}$ from the proposed method under the IND working correlation , EX correlation , and AR(1) correlation and from the method of ZP^35^ for data simulated under the cure rate 0.35 and the EX structure.

		(ζ,τ)=(0.4,0.8)				(ζ,τ)=(0.2,0.5)				(ζ,τ)=(0,0)			
		ZP	IND	AR(1)	EX	ZP	IND	AR(1)	EX	ZP	IND	AR(1)	EX
K=100 , n=3													
$\gamma_{0}$	Bias	0.015	0.014	0.014	0.013	−0.001	−0.003	−0.003	−0.003	0.008	0.006	0.006	0.007
	Var	0.044	0.044	0.042	0.039	0.036	0.036	0.036	0.035	0.032	0.031	0.031	0.031
	Var*	0.047	0.047	0.046	0.044	0.041	0.040	0.040	0.040	0.035	0.034	0.035	0.035
	CP	0.953	0.953	0.950	0.953	0.959	0.959	0.956	0.960	0.960	0.959	0.960	0.960
$\gamma_{1}$	Bias	0.017	0.015	0.012	0.013	0.025	0.023	0.026	0.023	0.023	0.020	0.019	0.019
	Var	0.083	0.083	0.069	0.065	0.071	0.071	0.070	0.067	0.077	0.076	0.077	0.076
	Var*	0.080	0.080	0.070	0.065	0.078	0.078	0.076	0.074	0.081	0.080	0.081	0.081
	CP	0.943	0.939	0.942	0.951	0.951	0.951	0.955	0.953	0.957	0.956	0.953	0.952
$\gamma_{2}$	Bias	0.023	0.020	0.013	0.014	0.022	0.019	0.018	0.017	0.027	0.023	0.023	0.024
	Var	0.069	0.068	0.064	0.058	0.061	0.060	0.059	0.057	0.066	0.066	0.066	0.065
	Var*	0.072	0.073	0.066	0.061	0.068	0.068	0.067	0.066	0.068	0.068	0.069	0.069
	CP	0.953	0.956	0.946	0.946	0.969	0.969	0.967	0.971	0.951	0.949	0.950	0.948
$\beta_{1}$	Bias	0.007	0.006	0.007	0.008	−0.002	0.001	0.003	0.004	−0.003	−0.001	−0.001	−0.001
	Var	0.028	0.021	0.014	0.013	0.025	0.019	0.017	0.016	0.027	0.020	0.020	0.020
	Var*	0.027	0.020	0.014	0.013	0.026	0.020	0.018	0.017	0.026	0.020	0.020	0.020
	CP	0.938	0.934	0.944	0.941	0.948	0.944	0.947	0.945	0.940	0.944	0.946	0.948
$\beta_{2}$	Bias	0.008	0.011	0.009	0.007	0.006	0.011	0.010	0.011	0.002	0.005	0.004	0.005
	Var	0.022	0.020	0.014	0.013	0.021	0.021	0.019	0.018	0.021	0.021	0.021	0.021
	Var*	0.022	0.021	0.016	0.014	0.021	0.021	0.019	0.018	0.020	0.020	0.020	0.020
	CP	0.950	0.946	0.963	0.959	0.936	0.936	0.937	0.938	0.940	0.943	0.943	0.946
K=60 , n=5													
$\gamma_{0}$	Bias	0.023	0.022	0.022	0.022	0.021	0.019	0.019	0.018	0.010	0.008	0.008	0.008
	Var	0.058	0.058	0.057	0.052	0.044	0.044	0.044	0.042	0.031	0.031	0.031	0.031
	Var*	0.060	0.061	0.061	0.056	0.046	0.047	0.047	0.046	0.034	0.034	0.034	0.034
	CP	0.955	0.955	0.954	0.955	0.958	0.959	0.958	0.960	0.956	0.955	0.954	0.955
$\gamma_{1}$	Bias	0.014	0.013	0.011	0.012	0.004	0.002	0.004	0.006	0.007	0.005	0.005	0.005
	Var	0.083	0.083	0.069	0.060	0.068	0.068	0.068	0.063	0.074	0.073	0.074	0.074
	Var*	0.082	0.083	0.075	0.063	0.078	0.078	0.077	0.072	0.076	0.076	0.077	0.077
	CP	0.937	0.940	0.951	0.951	0.959	0.962	0.956	0.963	0.952	0.953	0.952	0.951
$\gamma_{2}$	Bias	0.020	0.019	0.024	0.022	0.027	0.024	0.028	0.027	0.018	0.016	0.015	0.016
	Var	0.071	0.071	0.060	0.052	0.064	0.063	0.061	0.059	0.060	0.060	0.060	0.060
	Var*	0.077	0.078	0.072	0.060	0.068	0.069	0.069	0.065	0.066	0.066	0.067	0.067
	CP	0.959	0.958	0.957	0.957	0.947	0.951	0.953	0.951	0.951	0.953	0.955	0.955
$\beta_{1}$	Bias	−0.006	−0.004	−0.002	−0.004	0.001	0.002	0.002	0.003	0.001	0.002	0.001	0.001
	Var	0.025	0.019	0.012	0.010	0.024	0.018	0.016	0.015	0.025	0.019	0.019	0.019
	Var*	0.027	0.021	0.014	0.012	0.026	0.020	0.018	0.016	0.026	0.020	0.020	0.020
	CP	0.954	0.959	0.963	0.960	0.954	0.949	0.956	0.945	0.941	0.947	0.944	0.941
$\beta_{2}$	Bias	0.000	0.004	0.009	0.007	0.003	0.005	0.005	0.005	−0.004	0.001	0.001	0.001
	Var	0.024	0.024	0.015	0.013	0.021	0.021	0.019	0.018	0.019	0.018	0.019	0.019
	Var*	0.025	0.024	0.017	0.014	0.021	0.021	0.019	0.018	0.020	0.020	0.021	0.021
	CP	0.950	0.939	0.958	0.960	0.927	0.938	0.937	0.943	0.951	0.946	0.950	0.945

Var: empirical variance; Var*: average of bootstrap variance; CP: coverage percentage; IND: independent; EX: exchangeable; AR(1): first-order autoregressive; ZP: Zhang and Peng.

**Table 2.** Bias, Var, Var ${}^{*}$ , CP of 95% confidence intervals of ${\hat{\gamma }}_{0},{\hat{\gamma }}_{1},{\hat{\gamma }}_{2},{\hat{\beta }}_{1},{\hat{\beta }}_{2}$ from the proposed method under the IND working correlation , EX correlation , and AR(1) correlation and from the method of ZP^35^ for data simulated under the cure rate 0.5 and the EX structure.

		(ζ,τ)=(0.4,0.8)				(ζ,τ)=(0.2,0.5)				(ζ,τ)=(0,0)			
		ZP	IND	AR(1)	EX	ZP	IND	AR(1)	EX	ZP	IND	AR(1)	EX
K=100 , n=3													
$\gamma_{0}$	Bias	−0.002	−0.003	0.000	−0.001	−0.013	−0.014	−0.016	−0.016	−0.002	−0.004	−0.004	−0.003
	Var	0.050	0.050	0.047	0.046	0.038	0.037	0.037	0.037	0.034	0.034	0.034	0.034
	Var*	0.045	0.045	0.044	0.042	0.039	0.039	0.039	0.038	0.033	0.033	0.033	0.033
	CP	0.939	0.940	0.931	0.936	0.943	0.945	0.947	0.950	0.937	0.936	0.939	0.937
$\gamma_{1}$	Bias	0.009	0.008	0.003	0.004	0.025	0.023	0.029	0.027	0.015	0.014	0.014	0.014
	Var	0.067	0.067	0.059	0.056	0.071	0.071	0.070	0.069	0.069	0.069	0.070	0.069
	Var*	0.072	0.071	0.063	0.058	0.070	0.069	0.068	0.066	0.069	0.069	0.070	0.070
	CP	0.951	0.951	0.951	0.949	0.944	0.943	0.944	0.947	0.950	0.949	0.950	0.948
$\gamma_{2}$	Bias	0.017	0.016	0.020	0.018	0.020	0.018	0.017	0.016	0.009	0.006	0.006	0.007
	Var	0.059	0.059	0.051	0.047	0.056	0.056	0.056	0.054	0.055	0.055	0.055	0.056
	Var*	0.063	0.064	0.058	0.053	0.059	0.060	0.059	0.057	0.059	0.059	0.060	0.060
	CP	0.958	0.960	0.955	0.955	0.961	0.962	0.954	0.955	0.955	0.955	0.950	0.953
$\beta_{1}$	Bias	−0.009	−0.006	−0.003	−0.002	0.007	0.006	0.006	0.006	0.007	0.008	0.008	0.008
	Var	0.032	0.022	0.017	0.016	0.032	0.023	0.022	0.021	0.032	0.022	0.023	0.023
	Var*	0.036	0.026	0.020	0.018	0.034	0.024	0.023	0.022	0.034	0.024	0.025	0.026
	CP	0.958	0.959	0.961	0.959	0.952	0.955	0.953	0.953	0.952	0.948	0.955	0.951
$\beta_{2}$	Bias	−0.001	0.005	0.010	0.009	−0.007	−0.004	−0.003	−0.003	−0.001	0.001	0.001	0.002
	Var	0.030	0.028	0.020	0.019	0.024	0.025	0.024	0.023	0.028	0.028	0.029	0.029
	Var*	0.030	0.031	0.027	0.024	0.027	0.028	0.027	0.026	0.028	0.028	0.029	0.030
	CP	0.932	0.949	0.960	0.960	0.950	0.949	0.950	0.954	0.941	0.943	0.951	0.950
K=60 , n=5													
$\gamma_{0}$	Bias	0.006	0.005	0.005	0.004	0.004	0.003	0.003	0.001	0.003	0.002	0.001	0.002
	Var	0.052	0.052	0.053	0.049	0.044	0.044	0.043	0.043	0.030	0.030	0.030	0.030
	Var*	0.058	0.058	0.058	0.053	0.044	0.045	0.045	0.044	0.033	0.033	0.033	0.033
	CP	0.953	0.953	0.958	0.964	0.945	0.946	0.954	0.950	0.957	0.956	0.960	0.956
$\gamma_{1}$	Bias	0.013	0.012	0.009	0.013	0.016	0.015	0.016	0.019	0.017	0.014	0.015	0.014
	Var	0.069	0.069	0.060	0.050	0.069	0.069	0.066	0.064	0.063	0.062	0.062	0.062
	Var*	0.073	0.074	0.066	0.055	0.069	0.069	0.069	0.064	0.071	0.070	0.071	0.071
	CP	0.953	0.953	0.954	0.950	0.948	0.950	0.960	0.950	0.955	0.956	0.958	0.956
$\gamma_{2}$	Bias	0.024	0.024	0.020	0.021	0.032	0.031	0.029	0.030	0.029	0.026	0.026	0.026
	Var	0.062	0.062	0.055	0.047	0.051	0.051	0.051	0.047	0.055	0.055	0.055	0.055
	Var*	0.067	0.069	0.063	0.052	0.059	0.060	0.061	0.057	0.059	0.059	0.060	0.060
	CP	0.950	0.951	0.959	0.958	0.965	0.969	0.965	0.965	0.953	0.952	0.957	0.956
$\beta_{1}$	Bias	0.005	0.008	0.010	0.009	−0.003	0.001	0.000	0.000	0.009	0.007	0.005	0.006
	Var	0.034	0.026	0.019	0.016	0.033	0.024	0.022	0.020	0.033	0.023	0.023	0.023
	Var*	0.036	0.027	0.021	0.017	0.035	0.025	0.024	0.022	0.034	0.024	0.025	0.025
	CP	0.956	0.949	0.951	0.954	0.953	0.947	0.938	0.936	0.940	0.945	0.949	0.946
$\beta_{2}$	Bias	0.005	0.008	0.005	0.005	−0.004	0.001	0.001	0.003	0.013	0.015	0.014	0.013
	Var	0.033	0.031	0.023	0.020	0.027	0.029	0.027	0.025	0.027	0.028	0.028	0.028
	Var*	0.032	0.034	0.031	0.025	0.028	0.031	0.031	0.028	0.028	0.028	0.029	0.029
	CP	0.948	0.946	0.952	0.950	0.936	0.942	0.946	0.948	0.941	0.939	0.937	0.936

Var: empirical variance; Var*: average of bootstrap variance; CP: coverage percentage; IND: independent; EX: exchangeable; AR(1): first-order autoregressive; ZP: Zhang and Peng.

**Table 3.** Bias, Var, Var ${}^{*}$ , CP of 95% confidence intervals of ${\hat{\gamma }}_{0},{\hat{\gamma }}_{1},{\hat{\gamma }}_{2},{\hat{\beta }}_{1},{\hat{\beta }}_{2}$ from the proposed method under the IND working correlation , EX correlation , and AR(1) correlation and from the method of ZP^35^ for data simulated under the cure rate 0.35 and the AR(1) correlation structure.

		(ζ,τ)=(0.4,0.8)				(ζ,τ)=(0.2,0.5)				(ζ,τ)=(0,0)			
		ZP	IND	AR(1)	EX	ZP	IND	AR(1)	EX	ZP	IND	AR(1)	EX
K=100 , n=3													
$\gamma_{0}$	Bias	0.014	0.013	0.010	0.011	0.008	0.007	0.007	0.007	−0.003	−0.005	−0.004	−0.005
	Var	0.042	0.042	0.039	0.040	0.037	0.037	0.036	0.037	0.031	0.031	0.031	0.031
	Var*	0.044	0.044	0.041	0.042	0.039	0.039	0.038	0.039	0.034	0.034	0.034	0.034
	CP	0.953	0.954	0.954	0.954	0.949	0.947	0.946	0.947	0.958	0.958	0.958	0.958
$\gamma_{1}$	Bias	0.009	0.007	0.011	0.008	0.022	0.020	0.016	0.019	0.020	0.017	0.016	0.016
	Var	0.075	0.075	0.063	0.064	0.080	0.079	0.077	0.078	0.073	0.073	0.073	0.073
	Var*	0.080	0.079	0.068	0.070	0.078	0.078	0.076	0.077	0.077	0.077	0.077	0.077
	CP	0.952	0.954	0.957	0.958	0.936	0.937	0.941	0.940	0.951	0.951	0.955	0.953
$\gamma_{2}$	Bias	0.021	0.020	0.017	0.016	0.027	0.024	0.023	0.025	0.015	0.011	0.010	0.010
	Var	0.064	0.065	0.057	0.058	0.060	0.060	0.058	0.059	0.060	0.060	0.060	0.059
	Var*	0.070	0.071	0.062	0.063	0.068	0.068	0.066	0.067	0.065	0.065	0.066	0.066
	CP	0.959	0.958	0.954	0.960	0.963	0.963	0.965	0.963	0.954	0.956	0.956	0.959
$\beta_{1}$	Bias	0.007	0.007	0.008	0.007	0.009	0.009	0.007	0.008	0.011	0.009	0.009	0.009
	Var	0.026	0.020	0.013	0.014	0.024	0.018	0.016	0.017	0.027	0.020	0.020	0.020
	Var*	0.026	0.020	0.014	0.014	0.026	0.020	0.018	0.018	0.026	0.020	0.020	0.020
	CP	0.951	0.940	0.949	0.950	0.965	0.960	0.961	0.960	0.941	0.936	0.936	0.934
$\beta_{2}$	Bias	0.008	0.011	0.008	0.008	0.004	0.006	0.004	0.005	−0.003	−0.001	−0.001	−0.002
	Var	0.021	0.021	0.014	0.014	0.021	0.021	0.019	0.020	0.019	0.019	0.020	0.020
	Var*	0.022	0.021	0.015	0.015	0.021	0.021	0.019	0.019	0.020	0.020	0.020	0.020
	CP	0.946	0.949	0.951	0.957	0.944	0.942	0.936	0.936	0.942	0.943	0.939	0.943
K=60 , n=5													
$\gamma_{0}$	Bias	0.019	0.018	0.018	0.018	0.003	0.001	0.001	0.001	0.011	0.009	0.009	0.010
	Var	0.049	0.049	0.046	0.047	0.040	0.040	0.039	0.040	0.032	0.032	0.033	0.032
	Var*	0.048	0.048	0.044	0.046	0.039	0.039	0.039	0.039	0.034	0.034	0.034	0.034
	CP	0.944	0.943	0.940	0.949	0.946	0.945	0.950	0.946	0.947	0.946	0.947	0.945
$\gamma_{1}$	Bias	0.019	0.018	0.014	0.016	0.017	0.015	0.014	0.015	0.020	0.017	0.018	0.017
	Var	0.078	0.078	0.064	0.069	0.080	0.078	0.074	0.076	0.077	0.077	0.077	0.077
	Var*	0.079	0.080	0.066	0.073	0.081	0.080	0.076	0.078	0.078	0.078	0.079	0.078
	CP	0.952	0.954	0.941	0.951	0.951	0.950	0.953	0.951	0.949	0.950	0.951	0.947
$\gamma_{2}$	Bias	0.031	0.029	0.024	0.026	0.015	0.013	0.012	0.012	0.028	0.024	0.025	0.025
	Var	0.062	0.062	0.054	0.059	0.059	0.058	0.056	0.058	0.058	0.057	0.058	0.057
	Var*	0.071	0.072	0.061	0.066	0.067	0.067	0.065	0.067	0.066	0.067	0.067	0.067
	CP	0.957	0.957	0.956	0.958	0.964	0.962	0.966	0.967	0.965	0.965	0.968	0.964
$\beta_{1}$	Bias	0.001	0.005	0.004	0.007	−0.002	0.001	0.003	0.003	0.004	0.006	0.005	0.005
	Var	0.026	0.020	0.013	0.014	0.027	0.021	0.018	0.019	0.026	0.019	0.020	0.020
	Var*	0.026	0.020	0.013	0.015	0.026	0.020	0.017	0.018	0.026	0.020	0.020	0.020
	CP	0.937	0.943	0.955	0.954	0.930	0.929	0.938	0.945	0.939	0.935	0.942	0.938
$\beta_{2}$	Bias	−0.001	0.005	0.002	0.001	−0.005	0.001	0.001	0.000	−0.002	−0.002	−0.001	−0.002
	Var	0.021	0.021	0.014	0.015	0.021	0.022	0.018	0.019	0.019	0.018	0.018	0.019
	Var*	0.022	0.023	0.016	0.017	0.020	0.020	0.018	0.019	0.021	0.020	0.020	0.020
	CP	0.936	0.943	0.962	0.960	0.933	0.930	0.947	0.937	0.951	0.952	0.949	0.947

Var: empirical variance; Var*: average of bootstrap variance; CP: coverage percentage; IND: independent; EX: exchangeable; AR(1): first-order autoregressive; ZP: Zhang and Peng.

**Table 4.** Bias, Var, Var ${}^{*}$ , CP of 95% confidence intervals of ${\hat{\gamma }}_{0},{\hat{\gamma }}_{1},{\hat{\gamma }}_{2},{\hat{\beta }}_{1},{\hat{\beta }}_{2}$ from the proposed method under the IND working correlation , EX correlation , and AR(1) correlation and from the method of ZP^35^ for data simulated under the cure rate 0.5 and the AR(1) correlation structure.

		(ζ,τ)=(0.4,0.8)				(ζ,τ)=(0.2,0.5)				(ζ,τ)=(0,0)			
		ZP	IND	AR(1)	EX	ZP	IND	AR(1)	EX	ZP	IND	AR(1)	EX
K=100 , n=3													
$\gamma_{0}$	Bias	0.011	0.010	0.011	0.010	0.007	0.006	0.008	0.007	0.003	0.002	0.002	0.001
	Var	0.040	0.039	0.037	0.038	0.038	0.038	0.037	0.037	0.034	0.034	0.035	0.035
	Var*	0.043	0.043	0.040	0.041	0.037	0.038	0.037	0.037	0.033	0.033	0.033	0.033
	CP	0.962	0.963	0.963	0.962	0.944	0.945	0.948	0.954	0.938	0.940	0.934	0.934
$\gamma_{1}$	Bias	0.008	0.006	0.002	0.004	0.010	0.008	0.006	0.006	0.015	0.013	0.014	0.014
	Var	0.068	0.067	0.057	0.058	0.069	0.069	0.068	0.068	0.071	0.071	0.072	0.072
	Var*	0.072	0.072	0.061	0.063	0.069	0.069	0.067	0.068	0.069	0.069	0.070	0.070
	CP	0.961	0.961	0.958	0.961	0.948	0.947	0.940	0.946	0.943	0.940	0.944	0.942
$\gamma_{2}$	Bias	0.026	0.024	0.015	0.015	0.013	0.012	0.010	0.011	0.038	0.036	0.036	0.037
	Var	0.061	0.060	0.051	0.052	0.051	0.051	0.049	0.049	0.061	0.061	0.062	0.062
	Var*	0.062	0.063	0.053	0.055	0.059	0.060	0.059	0.059	0.059	0.060	0.061	0.061
	CP	0.946	0.948	0.952	0.948	0.963	0.962	0.961	0.967	0.951	0.949	0.949	0.951
$\beta_{1}$	Bias	0.006	0.003	0.003	0.003	−0.005	−0.002	0.000	−0.001	−0.012	−0.010	−0.011	−0.011
	Var	0.034	0.025	0.018	0.019	0.036	0.025	0.023	0.023	0.034	0.024	0.024	0.024
	Var*	0.034	0.025	0.019	0.019	0.034	0.024	0.023	0.023	0.034	0.024	0.025	0.025
	CP	0.953	0.951	0.955	0.952	0.937	0.945	0.943	0.946	0.941	0.937	0.946	0.947
$\beta_{2}$	Bias	0.012	0.012	0.007	0.008	−0.004	0.001	0.001	0.002	0.005	0.013	0.011	0.011
	Var	0.029	0.026	0.019	0.020	0.025	0.026	0.024	0.025	0.026	0.026	0.026	0.026
	Var*	0.029	0.029	0.023	0.023	0.028	0.029	0.028	0.028	0.028	0.028	0.029	0.029
	CP	0.943	0.949	0.957	0.956	0.954	0.954	0.958	0.957	0.958	0.956	0.957	0.959
K=60 , n=5													
$\gamma_{0}$	Bias	0.017	0.016	0.015	0.014	−0.004	−0.005	−0.001	−0.005	−0.013	−0.015	−0.015	−0.015
	Var	0.045	0.045	0.041	0.044	0.037	0.037	0.037	0.037	0.032	0.032	0.033	0.032
	Var*	0.046	0.047	0.043	0.045	0.038	0.039	0.038	0.039	0.033	0.033	0.033	0.033
	CP	0.946	0.946	0.956	0.948	0.954	0.957	0.953	0.952	0.948	0.950	0.948	0.948
$\gamma_{1}$	Bias	0.003	0.002	0.003	0.005	0.011	0.009	0.006	0.010	0.027	0.025	0.025	0.025
	Var	0.069	0.069	0.056	0.064	0.070	0.070	0.068	0.070	0.073	0.073	0.074	0.073
	Var*	0.071	0.072	0.059	0.065	0.069	0.069	0.067	0.068	0.070	0.070	0.071	0.071
	CP	0.946	0.948	0.950	0.955	0.951	0.949	0.939	0.940	0.939	0.939	0.940	0.939
$\gamma_{2}$	Bias	0.027	0.026	0.027	0.024	0.011	0.009	0.008	0.008	0.029	0.027	0.026	0.026
	Var	0.056	0.056	0.046	0.053	0.055	0.055	0.053	0.054	0.057	0.058	0.058	0.058
	Var*	0.063	0.065	0.054	0.060	0.058	0.059	0.058	0.059	0.059	0.059	0.060	0.060
	CP	0.957	0.961	0.964	0.960	0.948	0.952	0.952	0.950	0.958	0.957	0.954	0.956
$\beta_{1}$	Bias	0.007	0.008	0.008	0.009	0.002	0.002	0.002	0.001	−0.007	−0.004	−0.004	−0.005
	Var	0.037	0.027	0.019	0.021	0.032	0.023	0.022	0.023	0.032	0.023	0.023	0.023
	Var*	0.035	0.026	0.019	0.020	0.034	0.024	0.023	0.023	0.034	0.024	0.025	0.025
	CP	0.932	0.939	0.939	0.940	0.948	0.949	0.943	0.950	0.947	0.946	0.949	0.949
$\beta_{2}$	Bias	0.007	0.010	0.007	0.008	0.002	0.005	0.004	0.006	−0.004	0.000	0.000	−0.001
	Var	0.027	0.029	0.021	0.023	0.026	0.025	0.023	0.024	0.027	0.027	0.027	0.028
	Var*	0.029	0.031	0.026	0.026	0.027	0.029	0.029	0.028	0.028	0.028	0.029	0.029
	CP	0.944	0.948	0.955	0.952	0.941	0.958	0.949	0.951	0.933	0.944	0.939	0.941

Var: empirical variance; Var*: average of bootstrap variance; CP: coverage percentage; IND: independent; EX: exchangeable; AR(1): first-order autoregressive; ZP: Zhang and Peng.

For each setting above, we generate 1000 datasets and then fit each dataset with the proposed marginal model and the estimation method. As a comparison, we also fit the data with the AFT mixture cure model of Zhang and Peng^35^ (referred to as the ZP method) that does not consider the correlation within clusters. This method is available in the R package smcure.^47^ The average biases, empirical variances, average bootstrap variances (based on 100 bootstrap samples), and empirical coverage percentages of 95% confidence intervals of the estimators are reported in Tables 1 to 4. The results in the tables show that the average estimated variances of the regression parameters from the proposed method are close to their empirical variances in all cases, and the 95% confidence interval coverage rates are satisfactory and close to the nominal level. When the cure statuses and the failure times of uncured patients within a cluster are correlated, particularly for (ζ,τ)=(0.4,0.8) , the empirical variances of the regression parameters from the proposed method are less than those from the ZP method. When the working correlation structure in the proposed method coincides with the true correlation structure, the empirical variances are in general smaller than those with the misspecified one. For the same correlation strength, a higher cure rate generally implies smaller empirical variances in the incidence, especially for $\gamma_{1}$ and $\gamma_{2}$ , but larger variances in the latency, which is intuitive because in this case, fewer subjects are uncured. Given the same sample size, the empirical variance estimates tend to decrease as the correlation decreases. When the correlation reduces to zero, the biases and empirical variances based on the proposed method and the ZP method are comparable.

To further evaluate the efficiency gain of the proposed method, we calculate the relative efficiency defined as the ratio of mean squared errors (MSEs) of the estimates from the ZP method and the proposed method with the working independent, exchangeable, and first-order autoregressive correlation structures to the MSE of the estimates from the proposed method with the correctly specified working correlation structure. The results are summarized in Table 5, which shows that the MSEs from the proposed method with three working correlation structures are generally smaller than those from the ZP method when the values of (ζ,τ) are nonzero. That is, the proposed method improves the estimation efficiency potentially when the correlation exists. The MSEs from the proposed model with the correctly specified working correlation structure are less than those with a misspecified correlation structure, which indicates that using the correct working correlation structure can achieve higher efficiency, especially when the correlation within clusters is strong. The improvement in efficiency diminishes when the correlation within clusters becomes weak.

**Table 5.** Relative efficiencies (ratios of MSEs) from the proposed method under the IND working correlation, EX correlation, and AR(1) correlation and from the method of ZP^35^ against the proposed method under the correct correlation structure for data simulated under the cure rate 0.35 and 0.5 and the EX and the AR(1) correlation structures.

		(ζ,τ)=(0.4,0.8)				(ζ,τ)=(0.2,0.5)				(ζ,τ)=(0,0)			
(K,n)		ZP	IND	AR(1)	EX	ZP	IND	AR(1)	EX	ZP	IND	AR(1)	EX
Cure rate 0.35, EX correlation structure													
(100, 3)	${\hat{\gamma }}_{0}$	1.13	1.12	1.06	1.00	1.04	1.03	1.02	1.00	1.02	1.00	1.00	1.00
	${\hat{\gamma }}_{1}$	1.29	1.28	1.06	1.00	1.05	1.05	1.04	1.00	1.01	0.99	1.00	1.00
	${\hat{\gamma }}_{2}$	1.18	1.18	1.10	1.00	1.07	1.05	1.03	1.00	1.02	1.00	1.01	1.00
	${\hat{\beta }}_{1}$	2.12	1.64	1.09	1.00	1.54	1.17	1.03	1.00	1.33	0.99	1.00	1.00
	${\hat{\beta }}_{2}$	1.69	1.59	1.08	1.00	1.17	1.16	1.06	1.00	0.99	0.99	1.00	1.00
(60, 5)	${\hat{\gamma }}_{0}$	1.11	1.10	1.08	1.00	1.04	1.04	1.03	1.00	1.00	1.00	1.00	1.00
	${\hat{\gamma }}_{1}$	1.38	1.37	1.16	1.00	1.08	1.08	1.08	1.00	0.99	0.99	1.00	1.00
	${\hat{\gamma }}_{2}$	1.34	1.36	1.15	1.00	1.08	1.07	1.04	1.00	1.00	1.01	1.00	1.00
	${\hat{\beta }}_{1}$	2.45	1.91	1.19	1.00	1.59	1.22	1.07	1.00	1.28	0.98	0.99	1.00
	${\hat{\beta }}_{2}$	1.89	1.86	1.19	1.00	1.18	1.18	1.06	1.00	1.02	0.97	1.00	1.00
Cure rate 0.5, EX correlation structure													
(100, 3)	${\hat{\gamma }}_{0}$	1.09	1.09	1.03	1.00	1.02	1.02	1.01	1.00	1.00	1.00	1.00	1.00
	${\hat{\gamma }}_{1}$	1.21	1.21	1.06	1.00	1.03	1.02	1.02	1.00	1.00	1.00	1.00	1.00
	${\hat{\gamma }}_{2}$	1.26	1.26	1.09	1.00	1.03	1.04	1.03	1.00	0.99	0.99	1.00	1.00
	${\hat{\beta }}_{1}$	2.01	1.42	1.07	1.00	1.54	1.12	1.04	1.00	1.40	0.98	1.00	1.00
	${\hat{\beta }}_{2}$	1.57	1.43	1.07	1.00	1.05	1.09	1.02	1.00	0.96	0.98	1.00	1.00
(60, 5)	${\hat{\gamma }}_{0}$	1.07	1.07	1.09	1.00	1.02	1.02	1.00	1.00	1.00	1.00	0.99	1.00
	${\hat{\gamma }}_{1}$	1.37	1.38	1.18	1.00	1.08	1.09	1.03	1.00	1.01	0.99	0.99	1.00
	${\hat{\gamma }}_{2}$	1.32	1.34	1.17	1.00	1.08	1.09	1.08	1.00	0.99	0.99	0.99	1.00
	${\hat{\beta }}_{1}$	2.06	1.57	1.14	1.00	1.62	1.18	1.10	1.00	1.43	0.98	1.00	1.00
	${\hat{\beta }}_{2}$	1.61	1.53	1.12	1.00	1.07	1.17	1.09	1.00	0.97	0.99	1.01	1.00
Cure rate 0.35, AR(1) correlation structure													
(100, 3)	${\hat{\gamma }}_{0}$	1.06	1.06	1.00	1.02	1.01	1.01	1.00	1.01	0.99	0.99	1.00	1.00
	${\hat{\gamma }}_{1}$	1.20	1.19	1.00	1.02	1.04	1.03	1.00	1.01	1.00	1.00	1.00	1.00
	${\hat{\gamma }}_{2}$	1.13	1.14	1.00	1.01	1.04	1.03	1.00	1.01	1.00	1.00	1.00	0.99
	${\hat{\beta }}_{1}$	1.97	1.53	1.00	1.04	1.48	1.14	1.00	1.03	1.31	0.99	1.00	1.00
	${\hat{\beta }}_{2}$	1.55	1.51	1.00	1.03	1.11	1.10	1.00	1.03	0.98	0.99	1.00	1.00
(60, 5)	${\hat{\gamma }}_{0}$	1.08	1.08	1.00	1.03	1.04	1.03	1.00	1.02	1.00	1.00	1.00	1.00
	${\hat{\gamma }}_{1}$	1.22	1.21	1.00	1.08	1.08	1.05	1.00	1.03	1.00	0.99	1.00	1.00
	${\hat{\gamma }}_{2}$	1.15	1.15	1.00	1.09	1.05	1.04	1.00	1.03	1.00	0.98	1.00	0.99
	${\hat{\beta }}_{1}$	2.06	1.61	1.00	1.10	1.54	1.19	1.00	1.05	1.35	0.99	1.00	1.01
	${\hat{\beta }}_{2}$	1.56	1.55	1.00	1.09	1.21	1.22	1.00	1.09	1.03	0.99	1.00	1.01
Cure rate 0.5, AR(1) correlation structure													
(100, 3)	${\hat{\gamma }}_{0}$	1.07	1.06	1.00	1.02	1.02	1.02	1.00	1.01	0.99	0.99	1.00	1.01
	${\hat{\gamma }}_{1}$	1.20	1.18	1.00	1.02	1.02	1.02	1.00	1.01	0.99	0.99	1.00	1.01
	${\hat{\gamma }}_{2}$	1.19	1.17	1.00	1.02	1.04	1.04	1.00	1.00	0.99	0.99	1.00	1.00
	${\hat{\beta }}_{1}$	1.90	1.37	1.00	1.04	1.57	1.09	1.00	1.01	1.40	0.99	1.00	1.01
	${\hat{\beta }}_{2}$	1.51	1.35	1.00	1.02	1.03	1.06	1.00	1.02	1.00	1.00	1.00	1.01
(60, 5)	${\hat{\gamma }}_{0}$	1.10	1.10	1.00	1.07	1.02	1.01	1.00	1.01	0.99	0.99	1.00	1.00
	${\hat{\gamma }}_{1}$	1.24	1.24	1.00	1.15	1.03	1.03	1.00	1.02	0.99	0.99	1.00	1.00
	${\hat{\gamma }}_{2}$	1.22	1.23	1.00	1.15	1.04	1.04	1.00	1.02	0.99	0.99	1.00	1.00
	${\hat{\beta }}_{1}$	1.91	1.40	1.00	1.08	1.49	1.09	1.00	1.06	1.40	0.99	1.00	1.01
	${\hat{\beta }}_{2}$	1.29	1.37	1.00	1.10	1.11	1.08	1.00	1.02	0.99	0.99	1.00	1.01

MSE: mean squared error; IND: independent; EX: exchangeable; AR(1): first-order autoregressive; ZP; Zhang and Peng.

The proposed estimation method also produces estimates of $\rho_{1}$ and $\rho_{2}$ , the correlation coefficients in the two working correlation matrices. Even though they do not necessarily correspond to the correlation measures ζ and τ in the data generation, Table 6 shows that the estimated values of $\rho_{1}$ and $\rho_{2}$ agree well with the values of ζ and τ in the sense that when the latter decrease, the former tend to decrease too. When there is no correlation in clusters, the estimates of $\rho_{1}$ and $\rho_{2}$ are very close to zero. In other words, the estimated values of $\rho_{1}$ and $\rho_{2}$ provide good measures of the strength of the correlations between the cure statuses and between the failure times of uncured subjects in a cluster. The variance estimates of ${\hat{\rho }}_{1}$ and ${\hat{\rho }}_{2}$ are obtained based on the bootstrap method. We observe that the empirical variance estimates and the average of bootstrap variances are quite close, which indicates that the bootstrap variance estimator works well for calculating the variance estimates of ${\hat{\rho }}_{1}$ and ${\hat{\rho }}_{2}$ .

**Table 6.** Mean, Var, and the Var* of ( ${\hat{\rho }}_{1},{\hat{\rho }}_{2}$ ) based on the proposed method with the working EX or the AR(1) correlation structure.

	Working		(ζ,τ)=(0.4,0.8)			(ζ,τ)=(0.2,0.5)			(ζ,τ)=(0,0)		
(K,n)	correlation		Mean	Var	Var*	Mean	Var	Var*	Mean	Var	Var*
Cure rate 0.35, EX correlation structure											
(100, 3)	AR(1)	${\hat{\rho }}_{1}$	0.437	0.006	0.006	0.209	0.007	0.008	−0.006	0.005	0.005
		${\hat{\rho }}_{2}$	0.444	0.006	0.007	0.263	0.007	0.006	0.049	0.007	0.006
	EX	${\hat{\rho }}_{1}$	0.364	0.004	0.005	0.180	0.004	0.004	−0.005	0.003	0.003
		${\hat{\rho }}_{2}$	0.441	0.005	0.006	0.265	0.005	0.005	0.050	0.005	0.004
(60, 5)	AR(1)	${\hat{\rho }}_{1}$	0.540	0.009	0.009	0.237	0.011	0.010	−0.004	0.005	0.005
		${\hat{\rho }}_{2}$	0.445	0.006	0.007	0.261	0.006	0.006	0.050	0.006	0.005
	EX	${\hat{\rho }}_{1}$	0.363	0.004	0.004	0.176	0.004	0.003	−0.002	0.002	0.002
		${\hat{\rho }}_{2}$	0.443	0.005	0.005	0.260	0.004	0.004	0.049	0.003	0.003
Cure rate 0.5, EX correlation structure											
(100, 3)	AR(1)	${\hat{\rho }}_{1}$	0.439	0.006	0.006	0.210	0.007	0.007	−0.004	0.005	0.005
		${\hat{\rho }}_{2}$	0.374	0.006	0.007	0.225	0.006	0.007	0.077	0.008	0.007
	EX	${\hat{\rho }}_{1}$	0.366	0.004	0.004	0.180	0.004	0.004	−0.003	0.003	0.003
		${\hat{\rho }}_{2}$	0.373	0.004	0.005	0.225	0.005	0.005	0.077	0.006	0.005
(60, 5)	AR(1)	${\hat{\rho }}_{1}$	0.548	0.007	0.008	0.241	0.012	0.010	−0.004	0.004	0.005
		${\hat{\rho }}_{2}$	0.371	0.007	0.007	0.233	0.006	0.006	0.076	0.008	0.006
	EX	${\hat{\rho }}_{1}$	0.369	0.004	0.004	0.179	0.004	0.003	−0.005	0.002	0.002
		${\hat{\rho }}_{2}$	0.371	0.005	0.005	0.229	0.004	0.004	0.076	0.005	0.004
Cure rate 0.35, AR(1) correlation structure											
(100, 3)	AR(1)	${\hat{\rho }}_{1}$	0.370	0.005	0.005	0.180	0.006	0.005	0.001	0.005	0.005
		${\hat{\rho }}_{2}$	0.441	0.006	0.006	0.260	0.005	0.006	0.046	0.007	0.006
	EX	${\hat{\rho }}_{1}$	0.293	0.005	0.005	0.130	0.004	0.004	−0.002	0.004	0.003
		${\hat{\rho }}_{2}$	0.397	0.005	0.005	0.218	0.004	0.005	0.048	0.005	0.004
(60, 5)	AR(1)	${\hat{\rho }}_{1}$	0.367	0.005	0.005	0.179	0.005	0.005	−0.003	0.004	0.004
		${\hat{\rho }}_{2}$	0.442	0.006	0.006	0.265	0.005	0.005	0.045	0.006	0.005
	EX	${\hat{\rho }}_{1}$	0.200	0.004	0.004	0.082	0.003	0.002	−0.004	0.002	0.002
		${\hat{\rho }}_{2}$	0.336	0.004	0.005	0.174	0.004	0.003	0.046	0.004	0.003
Cure rate 0.5, AR(1) correlation structure											
(100, 3)	AR(1)	${\hat{\rho }}_{1}$	0.376	0.005	0.005	0.185	0.005	0.005	−0.002	0.006	0.005
		${\hat{\rho }}_{2}$	0.374	0.006	0.006	0.234	0.006	0.006	0.078	0.008	0.007
	EX	${\hat{\rho }}_{1}$	0.298	0.004	0.004	0.133	0.004	0.004	−0.003	0.004	0.003
		${\hat{\rho }}_{2}$	0.335	0.005	0.005	0.201	0.005	0.005	0.077	0.007	0.005
(60, 5)	AR(1)	${\hat{\rho }}_{1}$	0.369	0.005	0.005	0.182	0.005	0.005	−0.004	0.004	0.004
		${\hat{\rho }}_{2}$	0.373	0.005	0.006	0.231	0.005	0.005	0.080	0.008	0.006
	EX	${\hat{\rho }}_{1}$	0.200	0.003	0.003	0.083	0.003	0.003	−0.003	0.002	0.002
		${\hat{\rho }}_{2}$	0.283	0.004	0.004	0.164	0.005	0.004	0.078	0.005	0.004

Var: empirical variance; Var*: average of bootstrap variance; EX: exchangeable; AR(1): first-order autoregressive.

## 4. Contralateral breast cancer analysis

We apply the proposed method to a dataset of contralateral breast cancer patients from the SEER database of the National Cancer Institute (https://seer.cancer.gov/data/). Patients with unilateral breast cancer diagnosed between 2005 and 2008 and followed for contralateral breast cancer cases, invasive ductal carcinoma of no special type (ICD-O-3: 8500/3), positive lymph node statuses, and positive histology are considered along with the following baseline covariates: radiation therapy, age at diagnosis, estrogen receptor (ER) status, progesterone receptor (PR) status, and the lymph node ratio (LNR) defined as the ratio between the number of positive lymph nodes and the total number of examined lymph nodes.^48^ Patients with these characteristics are extracted from the SEER Plus Data 17 Registries database using SEER*Stat 8.4.0.1 software.

**Figure 1.**
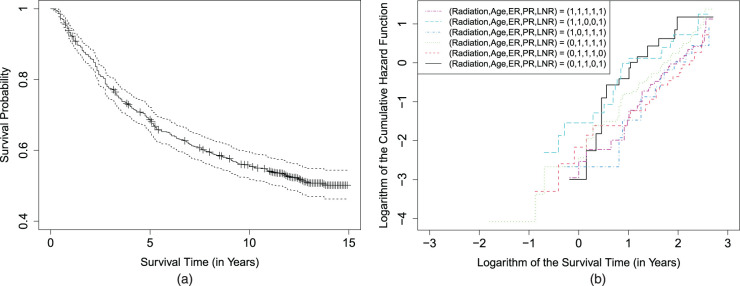
(a) The Kaplan–Meier survival curve and its pointwise 95% confidence interval and (b) the logarithm of estimated cumulative hazard functions of survival time distribution from some groups defined by the covariates in the cure model for the breast cancer data.

There are 694 eligible patients included in our study with a censoring rate of 52.6%. The survival time of interest is defined as the time (in years) to relapse or death due to the cancer. We plot the Kaplan–Meier survival curve in Figure 1(a) and observe that the survival curve presents a high plateau and levels off at a value substantially greater than 0 after 10 years of follow-up due to a large number of long-term survivors of the cancer. It indicates that the long-term survivors may be considered cured since they are unlikely to relapse or die of the cancer. We also conduct a nonparametric test^49^ for the existence of long-term survivors and the obtained p -value <0.05 shows significant evidence for the existence of cured or long-term survivors. Therefore, it is appropriate to consider a cure model for the data.

The 694 patients are from 44 clusters formed by SEER registries. Patients from the same cluster may share a similar lifestyle, a similar socioeconomic status, and a similar healthcare system, and their cure statuses and failure times of uncured patients may tend to be correlated due to the shared environments. Due to the number of clusters in the data, it will not be efficient to consider clusters as a categorical variable in the model. Therefore, the proposed marginal mixture cure model is suitable for analyzing the data to take into account the potential correlation within clusters.

Since we do not have any a priori subject knowledge about which of the baseline covariates should be in which part of the cure model, we consider all of them in both parts. The covariates are denoted and coded as follows: Radiation (a binary covariate with value 1 if a patient received radiation therapy and 0 otherwise), Age (a binary covariate with value 1 if a patient’s age is more than or equal to 50 years old and 0 otherwise), ER (a binary covariate with value 1 if a patient’s ER status is positive and 0 otherwise), PR (a binary covariate with value 1 if a patient’s PR status is positive and 0 otherwise), and LNR (the LNR between 0 and 1).

To examine the suitability of the AFT assumption and the PH assumption in the marginal mixture cure model for the data, following the idea of Zhang and Peng,^35^ we plot the logarithm of the cumulative hazard functions obtained from the weighted Kaplan–Meier survival estimators for the groups determined by the five covariates considered (LNR is dichotomized at 0.15^50^). The weight is estimated by $g_{ij}$ in (4) so that the weighted Kaplan–Meier survival estimator can be viewed as an estimator of the distribution of uncured subjects in the groups. If the PH assumption holds for the covariates in the latency part, then the logarithm of the estimated cumulative hazard functions from the groups should be parallel to each other. Figure 1(b) shows the logarithm of the estimated cumulative hazard functions for some groups. It is obvious that many of them are not parallel to each other substantially, indicating that the PH assumption for the uncured patients may not be appropriate. Furthermore, we follow the method of Peng and Taylor^51^ to calculate the Cramér–von Mises criterion based on the modified Cox–Snell residuals with all the covariates for both the fitted semiparametric PH mixture cure model and the semiparametric AFT mixture cure model by ignoring the clusters (the clusters should have no impact on the effects of covariates in the marginal model) and obtain the corresponding values $3.68\times 10^{-4}$ and $4.12\times 10^{-6}$ , respectively. The smaller value, which indicates a better fit to the data, suggests that the marginal semiparametric AFT mixture cure model is a better choice than the PH-based model for the data.

We fit the proposed model to the data under two working correlation structures: the working independent correlation and the working exchangeable correlation structures. As a comparison, we also fit the data with the AFT mixture cure model using the ZP method. The standard errors of estimates from the models are obtained using 100 bootstrap samples. The results from the models, summarized in Table 7, show some substantial differences. For example, in the latency part, we observe that Radiation is significant ( p -value = 0.047) under the proposed method with the working exchangeable correlation structure instead of marginal significant with the working independent correlation structure ( p -value = 0.052) and under the ZP method ( p -value = 0.093). This finding is perhaps due to the strong correlation ( ${\hat{\rho }}_{2}=0.313$ ) among the survival times of susceptible subjects within clusters revealed by the proposed method with the working exchangeable correlation structure. LNR is significant in both the incidence and the latency from all methods, which indicates that the higher the LNR, the lower the probability of being cured and the earlier events for uncured patients. Both ER and PR are significant in the latency for all methods, which implies that the uncured patients with either positive ER or positive PR tend to have later events. The results in the incidence part from the three methods are generally similar because of the weak correlation ( ${\hat{\rho }}_{1}=-0.013$ ) among the cure statuses of subjects within clusters.

**Table 7.** Estimated parameters and their SEs from the ZP method and from the proposed marginal semiparametric AFT mixture cure model under the working IND correlation and the working EX correlation structures for the breast cancer data.

	ZP			IND			EX		
	Estimate	SE	p -value	Estimate	SE	p -value	Estimate	SE	p -value
Incidence									
Intercept	−0.551	0.285	0.053	−0.584	0.256	0.023	−0.598	0.252	0.018
Radiation	−0.079	0.338	0.816	−0.065	0.304	0.831	−0.105	0.326	0.746
Age	0.162	0.222	0.465	0.169	0.206	0.411	0.154	0.207	0.457
ER	0.219	0.340	0.520	0.181	0.328	0.582	0.203	0.343	0.555
PR	−0.094	0.416	0.821	−0.183	0.370	0.621	−0.233	0.379	0.539
LNR	1.853	0.518	<0.001	1.872	0.490	<0.001	1.942	0.478	<0.001
${\hat{\rho }}_{1}$	–	–	–	–	–	–	−0.013	0.015	0.392
Latency									
Radiation	0.321	0.191	0.093	0.367	0.189	0.052	0.314	0.158	0.047
Age	−0.147	0.130	0.258	−0.137	0.128	0.284	−0.168	0.125	0.179
ER	0.483	0.134	<0.001	0.468	0.126	<0.001	0.538	0.124	<0.001
PR	0.545	0.197	0.006	0.467	0.181	0.010	0.407	0.155	0.009
LNR	−0.944	0.183	<0.001	−0.853	0.179	<0.001	−0.753	0.170	<0.001
${\hat{\rho }}_{2}$	–	–	–	–	–	–	0.313	0.066	<0.001

SE; standard error; AFT: accelerated failure time; ZP: Zhang and Peng; EX: exchangeable; IND: independent; ER: estrogen receptor; PR: progesterone receptor; LNR: lymph node ratio.

## 5. Conclusion and discussion

Marginal cure models have been widely used for analyzing multivariate survival data with a cure fraction. However, most efforts have been focused on the marginal PH mixture cure models which may be improper for the applications when the PH assumption is not satisfied for the latency. In this paper, we considered a semiparametric marginal AFT mixture cure model for correlated survival data with a cure proportion. A novel estimation approach is developed based on the GEE in the marginal AFT mixture cure model. We showed that the regression estimators are consistent and asymptotically normal, and employed a bootstrap method to estimate the variances of the estimated parameters. Our work relaxes the independent observations assumption for potentially correlated survival data on the usage of the AFT mixture cure model^35^ by incorporating working correlation structures in the estimation procedure. The proposed method is also an extension of the marginal AFT model,^9^ which was proposed for correlated failure time data without a cure fraction.

Our numerical studies show that the proposed method for clustered survival data improves the estimation efficiency of the regression estimators compared with the method in Zhang and Peng,^35^ especially when the correlation is strong and the cluster size is large. When the prespecified working correlation structure matches the underlying true correlation structure, the empirical variances are in general smaller than those under other working correlation structures. The results from the proposed method are comparable with the results from the method in Zhang and Peng,^35^ when the correlation strength declines or the cluster size decreases. Hence, the proposed semiparametric marginal AFT mixture cure model provides a new approach for the analysis of correlated lifetime data with a cure proportion, particularly when one is interested in characterizing covariate effects on the failure times of uncured patients directly, and the dependence among the survival times of uncured patients and among the cure statuses can be described by a few unknown parameters. The proposed model and estimation method also facilitate for future development of causal inference for clustered survival data with a cured fraction.

It is worth noting that although the models with random effects or frailties are often considered for clustered data where the random effects/frailties formulate the underlying dependence in a cluster, for clustered survival data with a cure fraction, we are not aware of any existing work based on the AFT assumption. Therefore, as pointed out by one reviewer, considering the AFT mixture cure model with random effects/frailties for clustered survival data should be an important and interesting topic for future research. However, a random effects/frailty model may not always be written as a marginal model unless under special cases such as a linear model with normal random effects. Thus, the estimates from a marginal model are generally not comparable with those from a random effects/frailty model.^52^

The bootstrap method is employed to estimate the variances of the estimators in the model in all the numerical work reported in this paper. This method is relatively straightforward to implement but can be computationally intensive. Having a computationally efficient method for variance estimation is always preferable to the bootstrap method.^53^ One interesting work in the future is to explore possibilities to simplify the variance estimation in the proposed method.

The proposed semiparametric estimation method for the marginal AFT mixture cure model was implemented in our R package smgeecure, which is publicly available at http://github.com/yiniu06/smgeecure.

## Appendix

Proof Proof of Theorem 1 The proof of the theorem is developed based on Rosen et al.^54^ and Liang and Zeger.^8^ We begin by listing the assumed conditions required for the consistency and asymptotic normality of $\hat{\theta }$ .

(C1) The censoring mechanism is noninformative.
(C2) The covariate vectors $X_{ij}$ and $Z_{ij}$ have bounded support. The design matrix formed by the column vectors of $X_{ij}$ and $Z_{ij}$ are of full rank.
(C3) With probability one, there exists a positive constant $\zeta_{0}$ such that $P(C_{ij}e^{-\beta_{0}^{\prime }Z_{ij}}\geq e^{\varepsilon_{ij}}\geq \tau_{0}|Z_{ij},X_{ij})>\zeta_{0}$ for all possible values of $Z_{ij}$ and $X_{ij}$ , where $\tau_{0}$ is a finite positive number.
(C4) For each fixed θ∈Θ , Ψ(θ) is $F_{t}-$ measurable, and Ψ(θ) is separable.
(C5) The function Ψ is a.s. continuous in θ : $\lim_{\theta^{\prime }\to \theta }|\Psi (\theta^{\prime })-\Psi (\theta )|=0,a.s.$
(C6) The expected value φ(θ)=E(Ψ(θ)) exists for all θ∈Θ , and has a unique zero at $\theta =\theta_{0}$ .
(C7) There exists a continuous function which is bounded away from zero, $b(\theta )\geq b_{0}>0$ , such that
  (i) $\underset{\theta }{\sup}|\Psi (\theta )|/b(\theta )$ is integrable,
  (ii) $\lim\underset{\theta \to \infty }{\inf}|\varphi (\theta )|/b(\theta )\geq 1$ ,
  (iii) $E\left\{\lim\underset{\theta \to \infty }{\sup}|\Psi (\theta )-\varphi (\theta )|/b(\theta )\right\}<1$ .
(C8) There are strictly positive numbers $a,b,c,d_{0}$ such that
  (i) $|\varphi (\theta )|>a\cdot |\theta -\theta_{0}|$ for $|\theta -\theta_{0}|\leq d_{0}$ ,
  (ii) $E\{\underset{|\tau -\theta |\leq d}{\sup}|\Psi (\tau )-\Psi (\theta )|\}<b\cdot d$ for $|\theta -\theta_{0}|+d\leq d_{0}$ , d≥0 ,
  (iii) $E[\{\underset{|\tau -\theta |\leq d}{\sup}|\Psi (\tau )-\Psi (\theta )|{\}}^{2}]\leq c\cdot d$ for $|\theta -\theta_{0}|+d\leq d_{0}$ , d≥0 .
(C9) The expectation $E\{|\Psi (\theta_{0}){|}^{2}\}$ is finite.

Let $\left(S_{t},F_{t},\sigma \right)$ , $\left(S_{c},F_{c},\mu \right)$ , and $\left(S_{\omega },F_{\omega },\nu \right)$ be σ -finite measure spaces, with a product measure space as follows: $(S_{t}⊗S_{c}⊗S_{\omega },F_{t}⊗F_{c}⊗F_{\omega },\sigma ⊗\mu ⊗\nu ),$ where $S_{t}\subset R^{d_{t}}$ , $S_{c}\subset R^{d_{c}},$ and $S_{\omega }\subset R^{d_{\omega }}$ . Here $d_{t}=\dim\;(t)$ , $d_{c}=\dim\;(c)$ , and $d_{\omega }=\dim\;(\omega )$ are defined as the dimension of the failure time t , the censoring time c , and the cure status w , respectively. We assume a marginal probability model $p_{ij}(t,c,\omega |\theta )$ for $(t_{ij},c_{ij},\omega_{ij})\in S_{t}⊗S_{c}⊗S_{\omega }$ , which is strictly positive on $S_{t}⊗S_{c}⊗S_{\omega }$ and may depend on i and j via covariates associated with the product measure σ⊗μ⊗ν for $i=1,\ldots ,K,j=1,\ldots ,n_{i}$ . Here θ is a vector-valued parameter in a subset Θ of $R^{d_{\theta }}$ with $d_{\theta }=\dim\;(\theta )$ , and $p_{ij}(t,c,\omega |\cdot )$ is continuously differentiable on Θ for each $(t_{ij},c_{ij},\omega_{ij})\in S_{t}⊗S_{c}⊗S_{\omega }$ . Let $p_{ij}(\omega |t_{ij},c_{ij},\theta )$ be the conditional probability model for all $\omega_{ij}\in S_{\omega }$ . That is, $p_{ij}(\omega |t_{ij},c_{ij},\theta )=p_{ij}(t_{ij},c_{ij},\omega |\theta )/\int_{S_{\omega }}p_{ij}(t_{ij},c_{ij},u|\theta )d\nu (u)$ . Let $q_{ij}(\cdot ,\cdot ,\cdot ;\cdot )$ be a $d_{\theta }\times 1$ vector-valued function composed by $\left\{U_{ij}^{\prime }(\gamma \right)$ , $U_{ij}^{\prime }(\beta ){\}}^{\prime }$ , where $U_{ij}(\gamma )$ and $U_{ij}(\beta )$ are the ij th summand of U(γ) and U(β) separately. We define $q_{ij}(\cdot ,\cdot ,\cdot ;\cdot )$ on $S_{t}⊗S_{c}⊗S_{\omega }⊗\Theta \mapsto R^{d_{\theta }}$ such that $q_{ij}(\cdot ,\cdot ,\cdot ;\varphi ):S_{t}⊗S_{c}⊗S_{\omega }\mapsto R^{d_{\theta }}$ is measurable and integrable with respect to $p_{ij}(\cdot ,\cdot ,\cdot |\theta )$ for each φ∈Θ , and $q_{ij}(t,c,w;\cdot ):\Theta \mapsto R^{d_{\theta }}$ is continuously differentiable on Θ for each $(t_{ij},c_{ij},\omega_{ij})\in S_{t}⊗S_{c}⊗S_{\omega }$ . We then define a bivariate function $H(\cdot |\cdot ):\Theta ⊗\Theta \mapsto R^{d_{\theta }}$ by $H(\varphi |\theta^{\left(m\right)})=\sum_{i=1}^{K}\sum_{j=1}^{n_{i}}\int_{S_{\omega }}q_{ij}(t_{ij},c_{ij},\omega ;\varphi )p_{ij}(\omega |t_{ij},c_{ij},\theta^{\left(m\right)})d\nu (\omega )$ . The E-step of the EM algorithm computes $H(\varphi |\theta^{\left(m\right)})$ , and the M-step solves for $\varphi =\theta^{\left(m+1\right)}$ from the equation $H(\varphi |\theta^{\left(m\right)})=0$ . As discussed by Niu and Peng,^17^ H(⋅|⋅) is a bivariate continuous function on Θ⊗Θ , where $\Theta \subseteq R^{d_{\theta }}$ . Next we prove that, $q_{ij}(\cdot ,\cdot ,\cdot ;\cdot )$ is an unbiased estimating function satisfying $E\{q_{ij}(t_{ij},c_{ij},y_{ij};\theta )|\theta \}=\iint \sum_{\omega }p_{ij}(\omega |t,c,\theta )q_{ij}(t,c,\omega ;\theta )dF_{t}(t|c,\theta )dF_{c}(c|\theta )=0$ for all θ∈Θ and all $j=1,\ldots ,n_{i}$ and i=1,…,K . In fact, proving the unbiasedness of $q_{ij}(\cdot ,\cdot ,\cdot ;\cdot )$ is equivalent to showing the unbiasedness of (9) and (10), that is, the conditional expectations of U(γ) and U(β) with respect to T are 0 . Since the unbiasedness of U(γ) can be established in the same way as in Niu and Peng,^17^ here we focus on the unbiasedness of estimating function U(β) , or correspondingly, $U_{ij}(\beta )$ . Let ${\tilde{Y}}_{ij}=\log\;{\tilde{T}}_{ij}$ which denotes the logarithm of the true underlying survival time. Following the idea of Buckley and James,^43^ we replace ${\tilde{Y}}_{ij}$ in the equation with its conditional expectation ${\hat{y}}_{ij}(\beta )$ evaluated at the regression coefficients β . By using the double-expectation formula and the Fubini’s theorem, we have

$$\begin{array}{rl} E\left\{U_{ij}(\beta )|c_{ij},\theta \right\} & =\sum_{\omega_{ij}}p_{ij}\left(\omega_{ij}|c_{ij},\theta \right)E\left\{U_{ij}(\beta )|\omega_{ij},c_{ij},\theta \right\}\\ & =\sum_{\omega_{ij}}p_{ij}\left(\omega_{ij}|c_{ij},\theta \right)M_{ij}\int \omega_{ij}\left({\hat{y}}_{ij}\left(\beta \right)-\beta^{\prime }X_{ij}\right)dF_{t}\left(t|\omega_{ij},c_{ij},\theta \right)\\ & =\pi_{ij}M_{ij}\int \left({\hat{y}}_{ij}\left(\beta \right)-\beta^{\prime }X_{ij}\right)dF_{t}\left(t|c_{ij},\theta \right)\\ & =\pi_{ij}M_{ij}\int \left(E\left({\tilde{Y}}_{ij}|t,c_{ij},\theta \right)-\beta^{\prime }X_{ij}\right)dF_{t}\left(t|c_{ij},\theta \right)\\ & =\pi_{ij}M_{ij}\left[\int E\left({\tilde{Y}}_{ij}|t,c_{ij},\theta \right)dF_{t}\left(t|c_{ij},\theta \right)-\beta^{\prime }X_{ij}\right]\\ & =\pi_{ij}M_{ij}\left[E\left({\tilde{Y}}_{ij}|c_{ij},\theta \right)-\beta^{\prime }X_{ij}\right]=\pi_{ij}M_{ij}\left[\beta^{\prime }X_{ij}-\beta^{\prime }X_{ij}\right]=0 \end{array}$$

where $\pi_{ij}=\pi (Z_{ij})$ , $M_{ij}=\phi_{2}^{-1}\sum_{l=1}^{n_{i}}(X_{il}-\bar{X})(\sigma_{\hat{Y}(\beta )}^{-1}{)}_{il}({\tilde{Q}}_{i}^{\left(2\right)}{)}_{lj}(\sigma_{\hat{Y}(\beta )}^{-1}{)}_{ij}$ and ${\tilde{Q}}_{i}^{\left(2\right)}=(Q_{i}^{\left(2\right)}{)}^{-1}$ . Then

$$\sum_{i=1}^{K}\sum_{j=1}^{n_{i}}E\{U_{ij}(\beta )|\theta \}=\sum_{i=1}^{K}\sum_{j=1}^{n_{i}}E\{E\{U_{ij}(\beta )|c_{ij},\theta \}\}=0$$

and we can obtain $\sum_{i=1}^{K}\sum_{j=1}^{n_{i}}E\{q_{ij}(t_{ij},c_{ij},\omega_{ij};\theta )|\theta \}=0$ . Therefore, following the proposition in Rosen et al.,^54^ if the algorithm converges, the final solution $\hat{\theta }$ satisfies the unbiased estimating equations $\Psi (\hat{\theta })=H(\hat{\theta }|\hat{\theta })=0$ . Furthermore, based on the regularity conditions, we assume at the beginning of the Appendix, the consistency and asymptotic normality of $\hat{\theta }$ follows from the result of Huber^55^ as the number of clusters K→∞ , which completes the proof of Theorem 1. Next we show the details of two items of Σ , that is, A(θ) and V(θ) , in the theorem. Obtaining V(θ) is straightforward. That is, we need to find $E\{S_{i}(\theta )\}=(E(U_{i}^{\prime }(\gamma )),E(U_{i}^{\prime }(\beta )){)}^{\prime }$ and calculate $V(\theta )=\Sigma_{i=1}^{K}E\{S_{i}(\theta )\}E\{S_{i}^{\prime }(\theta )\}$ . For A(θ) , it consists of two components, that is, E{B(θ)} and $E\{S(\theta )S^{\prime }(\theta )\}$ . The first component E{B(θ)} can be written as

$$E(B(\theta ))=-E\left\{\frac{\partial }{\partial \theta }S(\theta )\right\}=-E\left(\begin{array}{cc} U_{\gamma \gamma } & U_{\gamma \beta }\\ U_{\beta \gamma } & U_{\beta \beta } \end{array}\right)=-E\left(\begin{array}{cc} U_{\gamma \gamma } & 0\\ 0 & U_{\beta \beta } \end{array}\right)$$

The (h,k)(h,k=1,2,…,dim(γ)) element in the first dim(γ)×dim(γ) matrix $U_{\gamma \gamma }$ is

$$\sum_{i=1}^{K}A_{i}^{\left(\gamma \right)}\left[B_{i}^{\left(\gamma \right)}C_{i}^{\left(\gamma \right)}-D_{i}^{\left(\gamma \right)}E_{i}^{\left(\gamma \right)}\right]$$

where

$$\begin{array}{rl} A_{i}^{\left(\gamma \right)} & ={\left(Z_{i1h},Z_{i2h},\ldots ,Z_{in_{i}h}\right)}_{1\times n_{i}}\\ B_{i}^{\left(\gamma \right)} & ={\left(\begin{array}{ccc} B_{i11}^{\left(\gamma \right)} & \cdots & B_{i1n_{i}}^{\left(\gamma \right)}\\ \vdots & \ddots & \vdots \\ B_{in_{i}1}^{\left(\gamma \right)} & \cdots & B_{in_{i}n_{i}}^{\left(\gamma \right)} \end{array}\right)}_{n_{i}\times n_{i}} \end{array}$$

where $B_{imn}^{\left(\gamma \right)}=(1/2\phi_{1})(Z_{imk}(1-2\pi_{im})-Z_{ink}(1-2\pi_{in}))[\pi_{im}(1-\pi_{im}){]}^{1/2}[\pi_{in}(1-\pi_{in}){]}^{-1/2}({\tilde{Q}}_{i}^{\left(1\right)}{)}_{mn}$ for m≠n , otherwise $B_{imm}^{\left(\gamma \right)}=0,m,n=1,2,\ldots ,n_{i}$ , and ${\tilde{Q}}_{i}^{\left(1\right)}=(Q_{i}^{\left(1\right)}{)}^{-1}$ .

$$\begin{array}{rl} C_{i}^{\left(\gamma \right)} & =(\omega_{i1}-\pi_{i1},\omega_{i2}-\pi_{i2},\ldots ,\omega_{i2}-\pi_{i2}{)}^{\prime }\\ D_{i}^{\left(\gamma \right)} & ={\left(\begin{array}{ccc} D_{i11}^{\left(\gamma \right)} & \cdots & D_{i1n_{i}}^{\left(\gamma \right)}\\ \vdots & \ddots & \vdots \\ D_{in_{i}1}^{\left(\gamma \right)} & \cdots & D_{in_{i}n_{i}}^{\left(\gamma \right)} \end{array}\right)}_{n_{i}\times n_{i}} \end{array}$$

$D_{imn}^{\left(\gamma \right)}=\phi_{1}^{-1}[\pi_{im}(1-\pi_{im}){]}^{1/2}[\pi_{in}(1-\pi_{in}){]}^{-1/2}({\tilde{Q}}_{i}^{\left(1\right)}{)}_{mn},$ for m≠n , otherwise $D_{imm}^{\left(\gamma \right)}=({\tilde{Q}}_{i}^{\left(1\right)}{)}_{mm}$ .

$$E_{i}^{\left(\gamma \right)}=(Z_{i1k}\pi_{i1}(1-\pi_{i1}),Z_{i2k}\pi_{i2}(1-\pi_{i2}),\ldots ,Z_{in_{i}k}\pi_{in_{i}}(1-\pi_{in_{i}}){)}_{1\times n_{i}}^{\prime }$$

The second block diagonal dim(β)×dim(β) matrix $U_{\beta \beta }$ is

$$\begin{array}{rl} U_{\beta \beta } & =\frac{\partial }{\partial \beta }\left(\sum_{i=1}^{K}{\left(X_{i}-1_{i}{\bar{X}}^{\prime }\right)}^{\prime }{\left(B_{i}^{1/2}Q_{i}^{\left(2\right)}B_{i}^{1/2}\phi_{2}\right)}^{-1}G_{i}\left({\hat{Y}}_{i}\left(\beta \right)-X_{i}\beta \right)\right)\\ & =\sum_{i=1}^{K}{\left(X_{i}-1_{i}{\bar{X}}^{\prime }\right)}^{\prime }{\left(B_{i}^{1/2}Q_{i}^{\left(2\right)}B_{i}^{1/2}\phi_{2}\right)}^{-1}G_{i}\left\{\frac{\partial }{\partial \beta }{\hat{Y}}_{i}\left(\beta \right)-X_{i}\right\} \end{array}$$

where $(\partial /\partial \beta ){\hat{Y}}_{i}(\beta )=((\partial /\partial \beta ){\hat{y}}_{i1}(\beta ),\ldots ,(\partial /\partial \beta ){\hat{y}}_{in_{i}}(\beta ){)}^{\prime }$ and $\left(\partial /\partial \beta ){\hat{y}}_{ij}(\beta \right)$ is the first derivative of ${\hat{y}}_{ij}(\beta )$ with respect to β for i=1,…,K and $j=1,\ldots ,n_{i}$ . Specifically,

$$\begin{array}{rl} \frac{\partial }{\partial \beta }{\hat{y}}_{ij}\left(\beta \right) & =\frac{\partial }{\partial \beta }\left(\delta_{ij}Y_{ij}+\left(1-\delta_{ij}\right)\left[\frac{\int_{\varepsilon_{ij}\left(\beta \right)}^{\infty }ud{\hat{F}}_{\varepsilon }\left(u\right)}{1-{\hat{F}}_{\varepsilon }\left(\varepsilon_{ij}\left(\beta \right)\right)}+\beta^{\prime }X_{ij}\right]\right)\\ & =(1-\delta_{ij})\left[\frac{{\hat{f}}_{\varepsilon }(\varepsilon_{ij}(\beta ))X_{ij}{\hat{S}}_{\varepsilon }(\varepsilon_{ij}(\beta ))}{{\hat{S}}_{\varepsilon }^{2}(\varepsilon_{ij}(\beta ))}-\frac{\int_{\varepsilon_{ij}(\beta )}^{\infty }ud{\hat{F}}_{\varepsilon }(u){\hat{f}}_{\varepsilon }(\varepsilon_{ij}(\beta ))X_{ij}}{{\hat{S}}_{\varepsilon }^{2}(\varepsilon_{ij}(\beta ))}+X_{ij}\right]\\ & =(1-\delta_{ij})\left[{\hat{f}}_{\varepsilon }(\varepsilon_{ij}(\beta ))X_{ij}\frac{{\hat{S}}_{\varepsilon }(\varepsilon_{ij}(\beta ))-\int_{\varepsilon_{ij}(\beta )}^{\infty }ud{\hat{F}}_{\varepsilon }(u)}{{\hat{S}}_{\varepsilon }^{2}(\varepsilon_{ij}(\beta ))}+X_{ij}\right]\\ & =(1-\delta_{ij})\left[{\hat{h}}_{\varepsilon }(\varepsilon_{ij}(\beta ))X_{ij}\left(1-\frac{\int_{\varepsilon_{ij}(\beta )}^{\infty }ud{\hat{F}}_{\varepsilon }(u)}{{\hat{S}}_{\varepsilon }(\varepsilon_{ij}(\beta ))}\right)+X_{ij}\right]\\ & =(1-\delta_{ij})\left[{\hat{h}}_{\varepsilon }(\varepsilon_{ij}(\beta ))X_{ij}\left(1-{\hat{y}}_{ij}(\beta )\right)+X_{ij}\right] \end{array}$$

where ${\hat{h}}_{\varepsilon }(t)={\hat{f}}_{\varepsilon }(t)/{\hat{S}}_{\varepsilon }(t)$ , ${\hat{f}}_{\varepsilon }(t)=(\partial /\partial t){\hat{F}}_{\varepsilon }(t)$ and ${\hat{S}}_{\varepsilon }(t)=1-{\hat{F}}_{\varepsilon }(t)$ , which can be obtained based on (8). For the second component of A(θ) ,

$$E\left\{S\left(\theta \right)S^{\prime }\left(\theta \right)\right\}=E\left(\begin{array}{cc} U_{\gamma }U_{\gamma } & U_{\gamma }U_{\beta }\\ U_{\beta }U_{\gamma } & U_{\beta }U_{\beta } \end{array}\right)$$

where

$$\begin{array}{rl} E\left(U_{\gamma }U_{\gamma }\right) & =E{\left\{\sum_{i=1}^{K}R_{1i}^{\prime }V_{1i}^{-1}\left(\omega_{i}-g_{i}\right)\right\}}^{2}=\sum_{i=1}^{K}R_{1i}^{\prime }V_{1i}^{-1}E\left\{\left(\omega_{i}-g_{i}\right){\left(\omega_{i}-g_{i}\right)}^{\prime }\right\}(V_{1i}^{-1}{)}^{\prime }R_{1i}\\ E\left(U_{\gamma }U_{\beta }\right) & =E\left\{\sum_{i=1}^{K}R_{1i}^{\prime }V_{1i}^{-1}\left(\omega_{i}-g_{i}\right)\sum_{l=1}^{K}R_{2l}^{\prime }V_{2l}^{-1}(\omega_{l}-g_{l})({\hat{Y}}_{l}(\beta )-X_{l}\beta )\right\}\\ & =\sum_{i=1}^{K}R_{1i}^{\prime }V_{1i}^{-1}E\left\{\left(\omega_{i}-g_{i}\right)({\hat{Y}}_{i}(\beta )-X_{i}\beta {)}^{\prime }{\left(\omega_{i}-g_{i}\right)}^{\prime }\right\}(V_{2i}^{-1}{)}^{\prime }R_{2i} \end{array}$$

and

$$\begin{array}{rl} E(U_{\beta }U_{\beta }) & =E{\left\{\sum_{i=1}^{K}R_{2i}^{\prime }V_{2i}^{-1}\left(\omega_{i}-g_{i}\right)({\hat{Y}}_{i}(\beta )-X_{i}\beta )\right\}}^{2}\\ & =\sum_{i=1}^{K}R_{2i}^{\prime }V_{2i}^{-1}E\left\{\left(\omega_{i}-g_{i}\right)({\hat{Y}}_{i}(\beta )-X_{i}\beta )({\hat{Y}}_{i}(\beta )-X_{i}\beta {)}^{\prime }{\left(\omega_{i}-g_{i}\right)}^{\prime }\right\}(V_{2i}^{-1}{)}^{\prime }R_{2i} \end{array}$$

Here $\omega_{i}=(\omega_{i1},\ldots ,\omega_{in_{i}})$ , $R_{1i}=\partial \pi (Z_{i})/\partial \gamma$ , $V_{1i}=A_{i}^{1/2}Q_{i}^{\left(1\right)}A_{i}^{1/2}\phi_{1}$ , $R_{2i}=X_{i}-1_{i}{\bar{X}}^{\prime }$ and $V_{2i}=B_{i}^{1/2}Q_{i}^{\left(2\right)}B_{i}^{1/2}\phi_{2}$ .
